# Phytophthora Root Rot Modifies the Composition of the Avocado Rhizosphere Microbiome and Increases the Abundance of Opportunistic Fungal Pathogens

**DOI:** 10.3389/fmicb.2020.574110

**Published:** 2021-01-12

**Authors:** Itzel A. Solís-García, Oscar Ceballos-Luna, Elvis Marian Cortazar-Murillo, Damaris Desgarennes, Edith Garay-Serrano, Violeta Patiño-Conde, Edgar Guevara-Avendaño, Alfonso Méndez-Bravo, Frédérique Reverchon

**Affiliations:** ^1^Red de Estudios Moleculares Avanzados, Instituto de Ecología, A.C., Xalapa, Mexico; ^2^Escuela Nacional de Estudios Superiores Unidad Morelia, Laboratorio Nacional de Análisis y Síntesis Ecológica, Universidad Nacional Autónoma de México, Morelia, Mexico; ^3^Red de Biodiversidad y Sistemática, Instituto de Ecología, A.C., Xalapa, Mexico; ^4^CONACYT – Red de Diversidad Biológica del Occidente Mexicano, Instituto de Ecología, A.C., Pátzcuaro, Mexico; ^5^Instituto de Agroindustrias, Universidad Tecnológica de la Mixteca, Heroica Ciudad de Huajuapan de Leon, Mexico; ^6^CONACYT – Escuela Nacional de Estudios Superiores Unidad Morelia, Laboratorio Nacional de Análisis y Síntesis Ecológica, Universidad Nacional Autónoma de México, Morelia, Mexico; ^7^Red de Estudios Moleculares Avanzados, Instituto de Ecología, A.C., Pátzcuaro, Mexico

**Keywords:** *Fusarium*, *Mortierella*, *Persea americana*, rhizosphere microbial communities, root necrotizing fungi

## Abstract

The structure and function of rhizosphere microbial communities are affected by the plant health status. In this study, we investigated the effect of root rot on the avocado rhizosphere microbiome, using 16S rDNA and ITS sequencing. Furthermore, we isolated potential fungal pathogens associated with root rot symptoms and assessed their pathogenic activity on avocado. We found that root rot did not affect species richness, diversity or community structure, but induced changes in the relative abundance of several microbial taxa. Root rot increased the proportion of Pseudomonadales and Burkholderiales in the rhizosphere but reduced that of Actinobacteria, *Bacillus* spp. and Rhizobiales. An increase in putative opportunistic fungal pathogens was also detected in the roots of symptomatic trees; the potential pathogenicity of *Mortierella* sp., *Fusarium* spp., *Lasiodiplodia* sp. and *Scytalidium* sp., is reported for the first time for the State of Veracruz, Mexico. Root rot also potentially modified the predicted functions carried out by rhizobacteria, reducing the proportion of categories linked with the lipid and amino-acid metabolisms whilst promoting those associated with quorum sensing, virulence, and antibiotic resistance. Altogether, our results could help identifying microbial taxa associated to the disease causal agents and direct the selection of plant growth-promoting bacteria for the development of biocontrol microbial consortia.

## Introduction

The rhizosphere is a densely populated area influenced by the plant root exudates, where complex interactions occur between microbial communities and the radicular system (Yadav et al., 2015; Ahkami et al., 2017). The rhizosphere microbiota plays a fundamental role in plant growth, health, productivity, and in soil quality, as it may increase plant nutrient availability and uptake, abiotic stress tolerance, produce phytohormones and protect the plant against the attack of soil-borne pathogens (Bulgarelli et al., 2013; Mendes et al., 2013; Báez-Vallejo et al., 2020). Therefore, any shift in the composition of the rhizosphere microbiome could potentially affect its ecological functions, the physiological state of the plant and the plant productivity (Trivedi et al., 2012; Wei et al., 2018).

The structure and function of the rhizosphere microbial community are determined by the plant species, cultivar, the plant growth stage and the surrounding bulk soil (Berendsen et al., 2012; Nadarajah, 2016). Furthermore, the rhizosphere microbial composition is influenced by other abiotic and biotic factors that include soil physicochemical properties, climatic factors, agricultural practices, and the infection of the plant by a pathogen (Philippot et al., 2013; Lareen et al., 2016; Compant et al., 2019). An increasing number of studies have recently focused on the effects of disease on the composition of plant-associated microbial communities in economically important crops such as cotton, citrus, ginseng or tomato (Zhang et al., 2011; Trivedi et al., 2012; Wu et al., 2016; Kwak et al., 2018; Wei et al., 2018), reporting contrasting findings. Soil-borne pathogens, for example, can either induce an increase (Wu et al., 2016) or a decrease (Zhao et al., 2017) in microbial abundance in the rhizosphere. In the particular case of avocado (*Persea americana* Mill.), PCR-DGGE analysis showed that roots infected with *Phytophthora cinnamomi* were colonized by more diverse bacterial communities compared with those of healthy roots, which were colonized by a few predominant taxa (Yang et al., 2001). More recently, Shu et al. (2019) confirmed, using metagenomics, that avocado rhizosphere microbial communities were altered by root rot infection, as changes in relative abundance were observed within the top ten microbial taxa and several metabolic pathways were affected in bacteria and fungi. These results thus call for an in-depth analysis of the differences in the rhizosphere microbial community structure and composition caused by root-rot in avocado.

Avocado root rot represents the most devastating disease of the crop worldwide and the major limiting factor of avocado production in Australia, South Africa, California, and in some regions of Mexico where the disease has affected 50–90% of the orchards (Fernández-Pavía et al., 2013; Pagliaccia et al., 2013). The disease is primarily caused by the oomycete *Phytophthora cinnamomi*, although different microorganisms have also been reported as root rot causative agents in avocado trees, inducing the same symptomatology: necrosis of the feeder root system, occasional trunk cankers and ultimately, branch dieback (Zentmyer, 1980; Dann et al., 2012; Ploetz, 2013). Other identified root rot causal agents include several species of *Phytophthora* (Menge and Ploetz, 2003; Ploetz, 2013), as well as oomycetes and fungi such as *Cylindrocarpon* spp., *Fusarium* spp., *Nectria liriodendra*, *Ilyonectria macrodidyma* and *Pythium* sp. *amazonianum*, among others (Dann et al., 2011; Vitale et al., 2012; Carranza-Rojas et al., 2015; Ochoa et al., 2018). Such diversity of potential causal agents of root rot in avocado frequently hinders a correct diagnosis and an adequate management of the disease.

Mexico contributes to approximately 30% of the avocado global production, being the principal producer and exporter worldwide with an annual production of about 2.2 million tons (FAO., 2018). Although approximately 70% of the Mexican national production is concentrated in the temperate mountains of Michoacán State, in the Central-West part of the country, the cultivated area has rapidly expanded in the last few years throughout the whole territory (SIAP (Servicio de Información Agroalimentaria y Pesquera)., 2016). Avocado orchards are now being established under highly diverse environmental conditions, which enhances the risk of a high incidence of soil-borne necrotizing pathogens, especially in areas with high relative humidity and abundant rainfall. Therefore, it is important to study how root rot in avocado may perturb the rhizosphere microbial community and the functions it performs, as this would provide a better understanding of the complex microbial interactions existing in the rhizosphere. Studying the effect of avocado root rot on the rhizosphere microbiota is especially relevant in newly cultivated areas with high risk of soil-borne disease incidence.

Our previous studies have allowed us to identify microorganisms associated with root rot symptomatic and asymptomatic avocado trees with beneficial functions, such as pathogen-antagonistic and plant growth-promoting activities (Méndez-Bravo et al., 2018; Guevara-Avendaño et al., 2020). We have previously isolated and characterized bacterial strains with the ability to antagonize several avocado pathogens (Guevara-Avendaño et al., 2020) and to promote plant growth *in vitro* (Méndez-Bravo et al., 2018) by sampling an orchard with a high relative humidity in Veracruz State, Mexico. The objective of the present study was thus to characterize the impact of avocado root rot on the assembly of rhizosphere microbial community and predict shifts in their potential functions, using 16S ribosomal DNA (rDNA) and internal transcribed spacer (ITS) amplicon sequencing. Furthermore, we also used a culture-dependent approach to isolate potential fungal pathogens associated with root rot and assess their pathogenic activity on avocado. We hypothesized that root rot would modify the structure and diversity of rhizosphere microbial communities, and that avocado rotten roots could further attract opportunistic fungal pathogens, which would be reflected in the taxonomic composition of the fungal community.

## Materials and Methods

### Soil Sampling

Samples of rhizosphere soil of avocado were collected from the orchard San Carlos in Huatusco, Veracruz, as described in our previous studies (Méndez-Bravo et al., 2018; Guevara-Avendaño et al., 2020). A map of the orchard was provided in Méndez-Bravo et al. (2018). In the orchard, eight root rot asymptomatic avocado trees and eight root rot symptomatic avocado trees were selected, based on visual symptoms from root and leaf samples such as small, necrotic and brittle roots, chlorotic leaves and defoliation. The percentage of defoliated and wilted branches per tree, visually assessed as an indicator of the disease severity, ranged from 45 to 90%. All trees were planted at the same time and were therefore at the same growth stage (approximately 9 years old at the time of sampling). Root rot symptomatic trees were situated within an area susceptible to flooding, while asymptomatic trees were located on top of a hill, at a distance of approximately 200 m from the root-rot affected area. Trees within each sampling area were 5–20 m apart. For each tree, four rhizosphere soil samples of approximately 5 g were collected, according to the cardinal points, at 15–50 cm of the trunk and a depth of 5–10 cm, where the feeder roots were found, using a disinfected spade with 70% ethanol; samples were then homogenized into a single rhizosphere soil sample per tree (*n* = 8 per tree condition). Each rhizosphere soil sample consisted of fine roots and the soil around the roots. The soil samples were placed into Ziploc^®^ bags, labeled and maintained on ice, and immediately transported to the laboratory to be processed for DNA extraction or for isolation of culturable fungi.

### Soil DNA Extraction and Sequencing

Genomic DNA was extracted from 0.3 g of each rhizosphere soil sample using the DNeasy^®^ PowerSoil Kit (Qiagen, Germany), according to the manufacturer’s protocol. The DNA quantity and quality were checked on an Eppendorf BioSpectrometer^®^ (Eppendorf, Germany); DNA from one sample of the rhizosphere of a root rot symptomatic tree was degraded and therefore discarded.

For characterizing the bacterial communities, primers 341F (5′-CCTACGGGNGGCWGCAG-3′) and 805R (5′-GACTACHVGGGTATCTAATCC-3′) were used to amplify the V3 and V4 region of 16S rDNA gene (Herlemann et al., 2011; Qiu et al., 2020). 25 μL PCR reactions were performed with 40 ng μL^–1^ template DNA, 12.5 μL Multiplex Master Mix (Qiagen, Mexico), 1.25 μL each primer, 6 μL MilliQ water. PCR cycling parameters were as follows: 95°C for 15 min; 25 cycles at 95°C for 30 s, 50°C for 30 s, and 72°C for 30 s; and a final extension step at 72°C for 5 min. The amplicons were purified using the AMPure XP beads (Beckman Coulter, United States). Sequencing libraries were generated using the Nextera XT Index Kit (Illumina). Eight and seven libraries from the 16S rDNA amplicons were made for root rot asymptomatic and symptomatic avocado trees respectively (one per sampled tree). The libraries were sequenced in duplicate on the Illumina MiSeq platform 2 × 300 bp paired-end.

For fungal communities, genomic DNA was bulked in order to obtain one composite sample per tree condition (symptomatic vs. asymptomatic). Amplification of the ITS region was carried out with primers ITS1 (5′-TCCGTAGGTGAACCTGCGG-3′) and ITS4 (5′-TCCTCCGCTTATTGATATGC-3′). Amplification consisted of a first step at 95°C for 2 min; 25 cycles at 95°C for 30 s, 55°C for 30 s, 72°C for 30 s, and a final extension step at 72°C for 5 min. Two sequencing libraries per tree condition were generated from the amplicons of the ITS region, as described above. The four libraries were sequenced on four different lines on the Illumina NextSeq platform 2 × 150 bp paired-end. Data derived from bacterial and fungal sequencing were deposited in the Sequence Read Archive of NCBI under accession number PRJNA637654.

### Isolation of Culturable Fungi From Symptomatic Avocado Roots

In order to retrieve the potential fungal pathogens associated with root rot, fungal isolation was carried out from fine root samples collected from symptomatic avocado trees. Roots were washed and shaken in sterile water three times to eliminate soil particles. Surface disinfection of the roots was done by washing in 70% (v/v) ethanol for 1 min and rinsing with sterile water. After air-drying the surface of the roots under sterile conditions, the roots were cut into approximately 0.5–1 cm segments and placed onto Petri dishes with potato dextrose agar (PDA, Sigma-Aldrich), V8 juice agar media supplemented with chloramphenicol (150 mg L^–1^, Sigma-Aldrich) and peptone-agar amended with PCNB (100 μg mL^–1^), in triplicate. Plates were incubated in the dark at 24°C until fungal growth was observed.

Pure fungal cultures were obtained by transferring hyphal tips from the border of the actively growing colony onto fresh PDA or by single spore cultures as described in Choi et al. (1999). Fungal isolates were then grouped into morphotypes according to criteria such as macroscopic colony characteristics and microscopic hyphae, conidia and spore analyses by trypan blue staining. Conidial shape, color and size characterization was performed with 25 conidia from each representative isolate.

### DNA Extraction of Culturable Fungi and Sequencing

DNA extraction was carried out from one fungal isolate per morphotype (*n* = 10), following the CTAB protocol originally described by Wagner et al. (1987), which consists in grinding a sample of mycelium with a micro-pestle and treating it with proteinase K and extraction buffer. DNA amplification was performed with primers ITS1 and ITS4 following the parameters described in Tapia-Tussell et al. (2008). PCR amplicons were purified with the Wizard^®^ SV Gel and PCR Clean-Up System kit (Promega, United States) and sent to Macrogen Inc. (Seoul, South Korea) for Sanger sequencing. Sequences were edited in BioEdit 7.2.5 (Hall, 1999) and compared with the GenBank^1^ and Unite^2^ databases. The sequences and their best matches were aligned with MUSCLE in MEGA 7 (Kumar et al., 2016), and deposited in GenBank (accession numbers MT571538 to MT571547).

### Pathogenicity Tests

The pathogenic potential of the identified fungal isolates was assessed as described previously by Mayorquin et al. (2016), by conducting pathogenicity tests on healthy 2-year-old avocado plants (cv. Hass) acquired in a commercial nursery. The necrotizing ability of each isolate was tested in detached stems that were rinsed in distilled water and soaked in 10% sodium hypochlorite for 10 min. Leaves were removed and stems were cut into segments of approximately 30 cm long; the ends of the segments were generously covered with petroleum jelly to prevent desiccation. Each stem segment was wound-inoculated using a 4 mm-diameter cork borer to remove bark tissue in the middle part of the stem and introduce a mycelial plug from a 5-day-old culture. Sterile agar plugs were used as controls. Controls and inoculated wounds were covered with petroleum jelly, wrapped with cellophane, and subsequently placed in plastic containers with moistened paper towel at the bottom that were incubated at 24 °C. Necrotic progression was visually monitored every 7 days and after 4 weeks, stems were destructively sampled by removing bark and measuring the internal vascular necrosis. The disease incidence, expressed as percentage of symptomatic stems, was recorded and the necrotic damage (expressed as necrosis severity) was classified in a scale from 0 to 3, modified from Zentmyer, 1984; where: 0 = no symptoms except discoloration at the wound site; 1 = moderate vascular damage (necrotic lesions surrounding the site of inoculation); 2 = advanced damage (external necrosis, wilting and extended necrotic lesion through vascular tissue); and 3 = severe damage (generalized necrosis through the whole stem tissues). Five repetitions were carried out for each tested fungal isolate. Finally, necrotic tissue from the edge of lesions was sampled and cultured in PDA to morphologically identify the growing colonies and fulfill Koch’s postulates.

### Bioinformatic Analyses

For bacteria, sequences were quality-filtered using PRINSEQ v.0.20.40 (Schmieder and Edwards, 2011). Sequences with average quality scores < 20 and lengths < 100 bp were eliminated, no ambiguous bases (N) were conserved, and the reads were trimmed by 15 bases at the 5’-end and 3’-end (Jovel et al., 2016). In the quality filtering step, almost all reverse sequences were eliminated so only forward sequences were retained for the analysis. Chimeric sequences were removed using ChimeraSlayer (Haas et al., 2011). The obtained sequences were clustered into Operational Taxonomic Units (OTUs) by open-reference OTU picking (Caporaso et al., 2010b) at 97% sequence similarity using UCLUST (Edgar, 2010) and taxonomically assigned with a 0.8 confidence threshold using Greengenes database 13_8 (DeSantis et al., 2006; Kong et al., 2016). Chloroplast, mitochondria, archaea, singletons, unassigned sequences, low abundance OTUs (< 0.01%) and OTUs represented by 10 or fewer sequences in all samples were removed before further analysis (Lamelas et al., 2020). To construct the phylogenetic tree, reads were aligned using PyNAST (Caporaso et al., 2010a) and the Greengenes database, then the tree was made with FastTree (Price et al., 2010). For downstream analyses, the OTU sequence counts were normalized with two approaches, a relative abundance normalization, where the sequencing reads in a given sample were divided by the total number of sequencing reads in that sample (Fitzpatrick et al., 2018) and the normalization by rarefaction, where the sequences were randomly subsampled at the same sequence depth (19,784 sequences per sample) (Caporaso et al., 2010b). The OTU table normalized by relative abundance was used for the determination of shared OTUs using R (R Core Team, 2019) and the Venn diagram was constructed with SmartDraw.

The prediction of bacterial functional composition was made following the PICRUST pipeline v.1.1.4 (Langille et al., 2013), removing *de novo* OTUs in order to keep only those OTUs compatible with Greengene IDs (Caporaso et al., 2010a). The predicted functions table was classified into KEGG pathways at level 3.

For rhizosphere soil fungal communities, forward and reverse primers were removed and the paired-end sequences were merged when possible; typically, the forward reads were retained when the sequences did not merge. Reads with a minimum length of 100 pb were removed and the reads with a maximum length of 300 pb were trimmed, using the pre-processing script of AMPtk (Palmer et al., 2018). Quality filtering was performed after the merging of paired reads, with a maximum expected error of 2 using VSEARCH v2.7.1 (Edgar and Flyvbjerg, 2015; Rognes et al., 2016). The sequences were re-labeled by unique sample tags, joined into a single file and unique sequences were detected with the USEARCH algorithm v11.0.667 (Edgar, 2010). All the reads were clustered in OTUs at 97% sequence similarity, the chimeric sequences were removed, and the singletons were excluded from the dataset, using the UPARSE-OTU algorithm (Edgar, 2013). Taxonomic assignment was implemented using the hybrid taxonomy algorithm of AMPtk against the UNITE database v8.0 (Nilsson et al., 2019), which consists in calculating a consensus last common ancestor based on two successive alignments: 1) a global alignment and 2) an alignment to Bayesian Classifiers. The Amptk default threshold values were used for taxonomic assignment, as recommended in Palmer et al. (2018). Following taxonomic assignment, unassigned sequences and taxonomic levels not corresponding to fungi were removed. Using the same criteria as for bacteria, fungal OTUs with less than 0.01% relative abundance and represented by 10 or fewer sequences in all samples were removed. Shared OTUs between symptomatic and asymptomatic trees were obtained using R and were visualized with a Venn diagram that was designed in SmartDraw. The FUNGuild v1.0 database (Nguyen et al., 2016) was used to assign the trophic categories and ecological guild to each OTU, only the categories with probable and highly probable confidence rank were retained.

### Bacterial Community Diversity and Statistical Analyses

Rarefaction curves for bacterial and fungal data were created using the phyloseq, ranacapa, vegan and ggplot2 packages in R v.3.5.2 software. For bacterial communities, statistical analyses were computed in R v.3.5.2 software, using a significance value of *P* < 0.05. Bacterial richness and alpha diversity metrics (observed species richness, Shannon index and Simpson index) were calculated using the phyloseq package (McMurdie and Holmes, 2013) in R from the OTU table normalized by rarefaction, to minimize the influence of sequencing depth on the results (Weiss et al., 2017). Means and standard errors of richness and alpha diversity metrics were calculated with the package psych and the differences were tested with the Mann-Whitney-Wilcoxon test with a continuity correction. For beta diversity analyses, the OTU table normalized by relative abundance (Fitzpatrick et al., 2018) was log2-transformed and Unifrac weighted and unweighted dissimilarity matrices were computed using the phyloseq and vegan packages (Oksanen et al., 2018). Non-metric multidimensional scaling (NMDS) based on Unifrac weighted and unweighted distances were computed using the same packages. A permutational multivariate analysis of variance (PERMANOVA) was used to compare the structure of the bacterial rhizosphere community of root rot asymptomatic and symptomatic trees, using the vegan package with the Unifrac weighted and unweighted distance matrix with 999 permutations. Significant differences in bacterial taxa relative abundance between root rot asymptomatic and symptomatic trees were evaluated using a Mann-Whitney-Wilcoxon test with an FDR correction.

Statistical analysis of the bacterial metabolic profiles was performed with a White’s non-parametric *t*-test (White et al., 2009) and a multiple testing correction with the Benjamini-Hochberg method, using the software STAMP (Parks and Beiko, 2010).

For fungal communities, a Generalized Fold Change (GFOLD) analysis was carried out in order to assess differences in the abundance of fungal OTUs between asymptomatic and symptomatic trees. The GFOLD algorithm generates rankings of differentially abundant taxa from samples without biological replicates (Feng et al., 2012; Ibarra-Juárez et al., 2018) and thus allowed us to compare data from our composite samples. Read counts at each taxonomic level were normalized by the library size of each sample and the data was used to estimate the log2 fold change (log2fdc), considering values of log2fdc ≠ 0 and GFOLD(0.01) ≠ 0 as differentially abundant (Ibarra-Juárez et al., 2018).

## Results

### Diversity of Rhizosphere Bacterial and Fungal Communities

A total of 2,557,554 and 4,185,892 raw sequences were obtained from bacteria and fungi, respectively. After quality filtering, 1,163,164 high-quality reads from bacteria and 293,499 from fungi were obtained. These reads were clustered into 3,424 bacterial OTUs and 1,184 fungal OTUs at a 97% sequence similarity (Supplementary Table S1). Rarefaction curves showed that all rhizosphere soil samples from root rot asymptomatic and symptomatic avocado trees reached a plateau (Supplementary Figure S1), indicating that most OTUs from the rhizosphere bacterial and fungal communities were detected.

The numbers of OTUs were rarefied based on the library with the lowest number of reads, resulting in 3,411 bacterial OTUs and 1,184 fungal OTUs. For both bacterial and fungal communities, richness and alpha diversity values in the rhizosphere of root rot symptomatic trees were similar to those observed in the rhizosphere of asymptomatic trees (Table 1). Simpson’s indices presented large values for both bacteria and fungi, indicating the presence of dominant OTUs in the avocado rhizosphere microbial community.

**TABLE 1:** Richness and alpha diversity of rhizosphere bacterial and fungal communities associated with root rot asymptomatic and symptomatic avocado trees.

	**Observed OTUs**	**Shannon index (H’)**	**Simpson’s index (D)**
**Bacteria**			
Asymptomatic (*n* = 16)	888 ± 71	6.04 ± 0.04	0.99 ± 0.0001
Symptomatic (*n* = 14)	810 ± 82	5.92 ± 0.13	0.99 ± 0.003
**Fungi**			
Asymptomatic (*n* = 2)	545	4.56	0.97
Symptomatic (*n* = 2)	574	4.81	0.97

*Values represent the means ± SE. “n” represents the number of sequencing libraries. No significant differences were observed in bacterial alpha diversity metrics (P > 0.05, Mann-Whitney-Wilcoxon test).*

**FIGURE 1:**
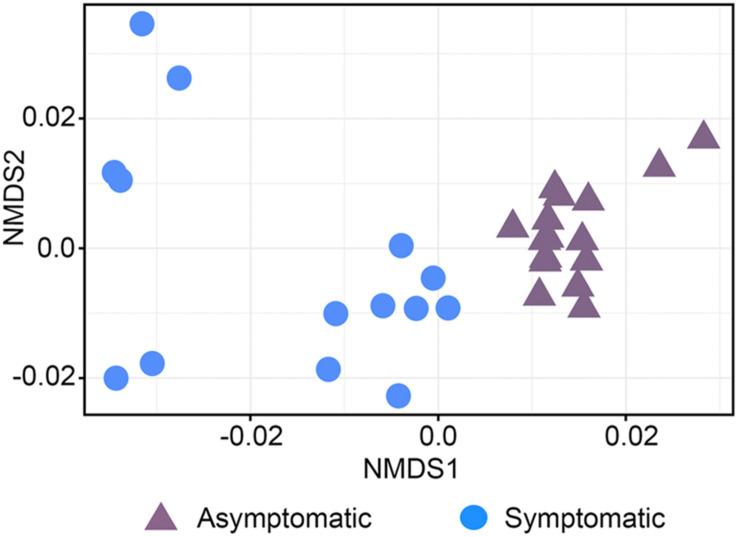
Non-metric multidimensional scaling (NMDS) plot based on UniFrac weighted distance of the bacterial community structure associated with the roots of root rot asymptomatic and symptomatic avocado trees (stress value = 0.10).

The beta diversity of the bacterial community was represented in a NMDS plot based on weighted UniFrac distances (stress value = 0.10, Figure 1). Although phylogenetic dissimilarities between samples showed that the rhizosphere bacterial community of asymptomatic trees was separately clustered from that of symptomatic trees, the PERMANOVA analysis revealed that the structure of the bacterial community between the two groups of trees was not significantly different (Supplementary Table S2, *F* = 1.10, *P* = 0.33). A similar pattern of clustering was observed in a NMDS plot using unweighted UniFrac distances (stress value = 0.15, Supplementary Figure S2).

### Composition of Rhizosphere Bacterial and Fungal Communities

**FIGURE 2:**
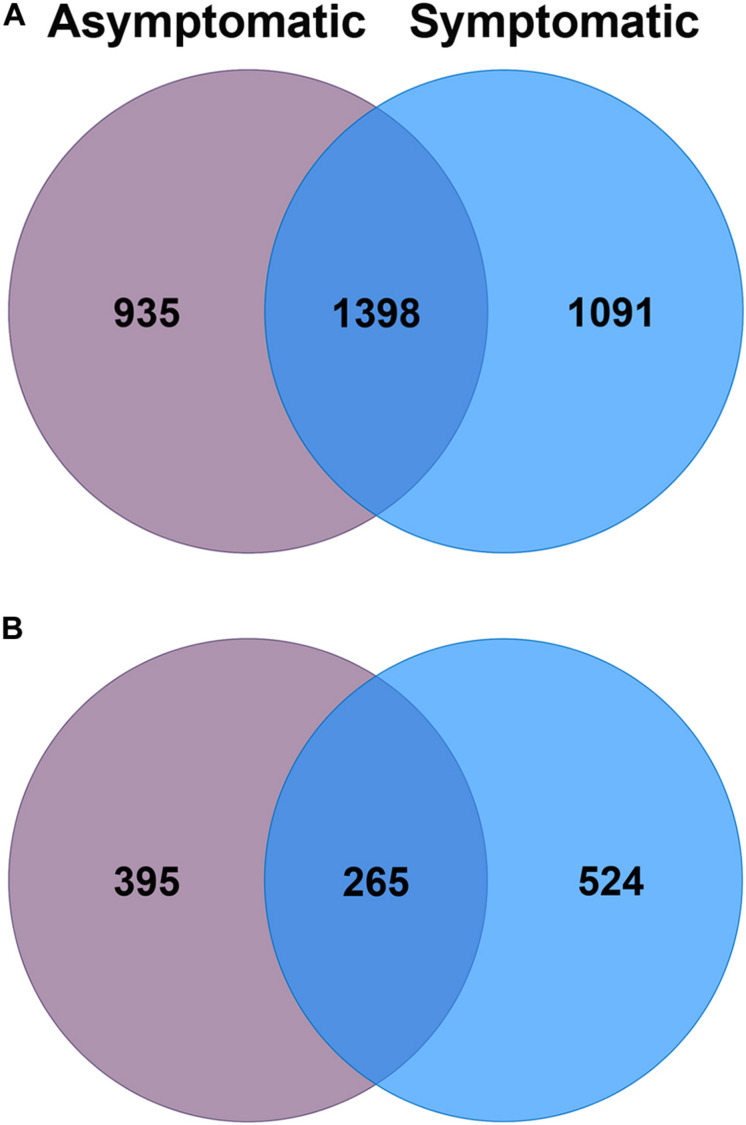
Venn diagrams of the number of unique and shared operational taxonomic units (OTUs) of the **(A)** bacterial and **(B)** fungal community associated with the rhizosphere of root rot asymptomatic (purple) and symptomatic (blue) avocado trees.

Of the total 3,424 bacterial OTUs that were detected in the avocado rhizosphere, 27% were exclusively found in root rot asymptomatic trees whilst 32% were only associated with root rot symptomatic trees (Figure 2). The avocado rhizosphere fungal community was represented by a total of 1,184 OTUs, of which 33% were exclusively detected in root rot asymptomatic trees whilst 44% were only found in root rot symptomatic trees. The Venn diagrams also showed that 41% of bacterial and 22% of fungal OTUs were shared between the two groups of trees (Figure 2).

**FIGURE 3:**
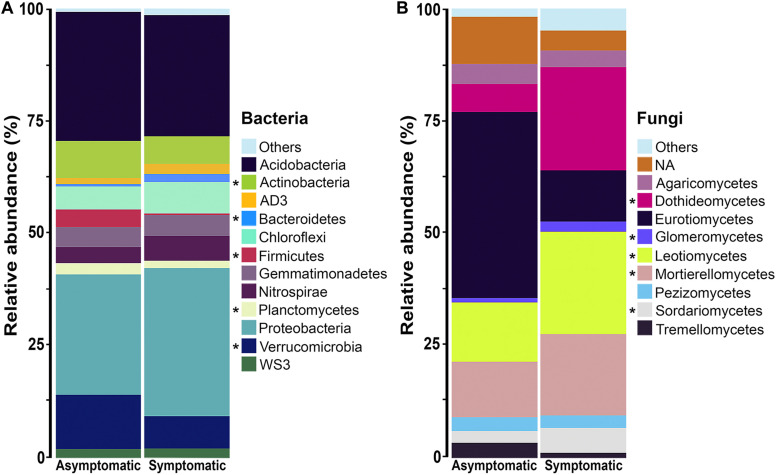
Phylum composition of the **(A)** bacterial and class composition of the **(B)** fungal community associated with the rhizosphere of root rot asymptomatic and symptomatic avocado trees. Low abundance taxonomic groups (relative abundance < 1%) were reported as Others. NA means not assigned. The asterisk indicates significant difference in taxa relative abundance between root rot asymptomatic and symptomatic trees (For bacteria: *P* < 0.05, Mann-Whitney-Wilcoxon test; for fungi: log2fdc ≠ 0 and GFOLD(0.01) ≠ 0, GFOLD algorithm).

The rhizosphere bacterial communities associated with root rot asymptomatic and symptomatic trees were dominated by phyla Proteobacteria, Acidobacteria, Verrucomicrobia, Actinobacteria, and Chloroflexi (Figure 3A). However, differences were observed in the relative abundance of various bacterial phyla between root rot symptomatic and asymptomatic trees (Supplementary Table S3). Phyla Verrucomicrobia (*W* = 209, *P* ≤ 0.05), Actinobacteria (*W* = 180, *P* ≤ 0.05), Firmicutes (*W* = 224, *P* ≤ 0.05) and Planctomycetes (*W* = 178, *P* ≤ 0.05) were significantly more abundant in the rhizosphere of root rot asymptomatic trees, whilst the relative abundance of Bacteroidetes (*W* = 35, *P* ≤ 0.05) was larger in that of symptomatic trees. The composition of the avocado rhizosphere bacterial community at lower taxonomic levels is shown in Supplementary Figure S3. Bacterial taxa such as Rhizobiales (*W* = 209, *P* ≤ 0.05) and Bacillales (*W* = 224, *P* ≤ 0.05) were more enriched in the bacterial community of root rot asymptomatic trees than in that of symptomatic trees. Conversely, the abundance of Pseudomonadales (*W* = 8, *P* ≤ 0.05) and Burkholderiales (*W* = 9, *P* ≤ 0.05) populations increased in the bacterial community of root rot symptomatic trees (Supplementary Tables S4, S5).

The rhizosphere fungal communities associated with root rot asymptomatic and symptomatic trees showed significant differences in the abundance of several taxa, according to the GFOLD algorithm (Supplementary Tables S6–S8). Phyla Ascomycota and Mortierellomycota were the most dominant in the rhizosphere of both root rot asymptomatic and symptomatic trees (Supplementary Figure S4A and Supplementary Table S6). At the class level, Eurotiomycetes, Agaricomycetes, Pezizomycetes and Tremellomycetes populations showed a differentially higher relative abundance in the rhizosphere of root rot asymptomatic trees, whilst Leotiomycetes, Mortierellomycetes, Dothideomycetes, Sordariomycetes and Glomeromycetes populations dominated the rhizosphere of root rot symptomatic trees (Figure 3B and Supplementary Table S7). Analyses at the order level showed that taxa such as Helotiales, Mortierellales, Venturiales and Pleosporales were significantly enriched in the rhizosphere of root rot symptomatic trees (Supplementary Figure S4B and Supplementary Table S8).

### Potential Functions of the Rhizosphere Bacterial and Fungal Community

**FIGURE 4:**
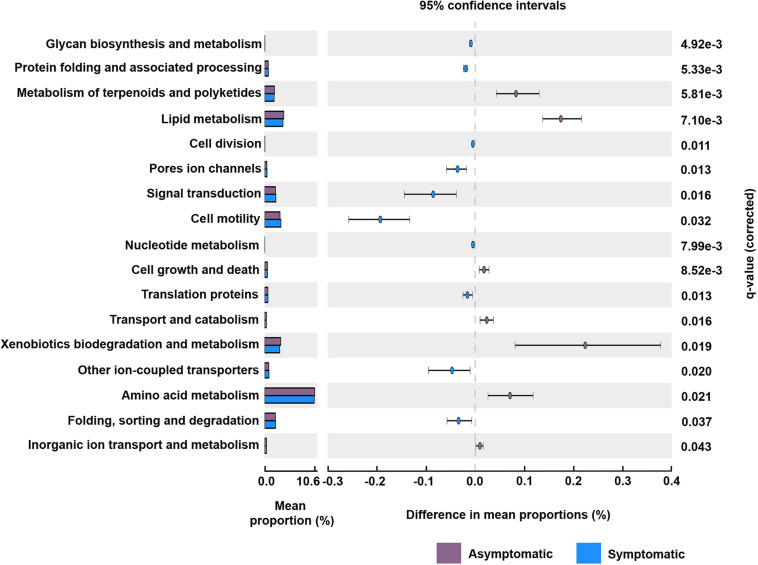
Potential functional gene categories of the rhizosphere bacterial community associated with root rot asymptomatic and symptomatic trees. Differences in the mean abundance of each category, at level 2 KEGG orthology (KO), are showed. The *q*-values were derived from a White’s non-parametric *t*-test with Benjamini-Hochberg correction.

The predicted functional analysis of the bacterial community was made from 1,919 (56%) OTUs. The predicted bacterial functional profiling revealed a higher abundance of sequences associated with metabolism in the rhizosphere of root rot asymptomatic avocado trees than in that of symptomatic trees, at the level 1 KEGG Orthology (KO) (*q* = 0.002). Furthermore, the functional category “cellular processes” was significantly more abundant in bacterial communities associated with root rot symptomatic avocado trees (*q* = 0.008) (Supplementary Figure S5). Based on the profiling data at level 2 KO, 17 principal functional gene categories were identified for bacteria in the rhizosphere of avocado trees (Figure 4). Some of the most represented categories in the rhizosphere of symptomatic avocado trees included signal transduction (*q* = 0.016) and cell motility (*q* = 0.032). These functional gene categories appear to be involved in various functions such as bacterial secretion system (*q* = 0.017), two-component system (*q* = 0.009), bacterial motility proteins (*q* = 0.008) and bacterial chemotaxis (*q* = 0.023) (Supplementary Figure S6). In the bacterial functional profile of the rhizosphere of asymptomatic trees, the amino acid metabolism (*q* = 0.021) and the metabolism of terpenoids and polyketides (*q* = 0.005) were some of the most enhanced functional gene categories (Figure 4).

**FIGURE 5:**
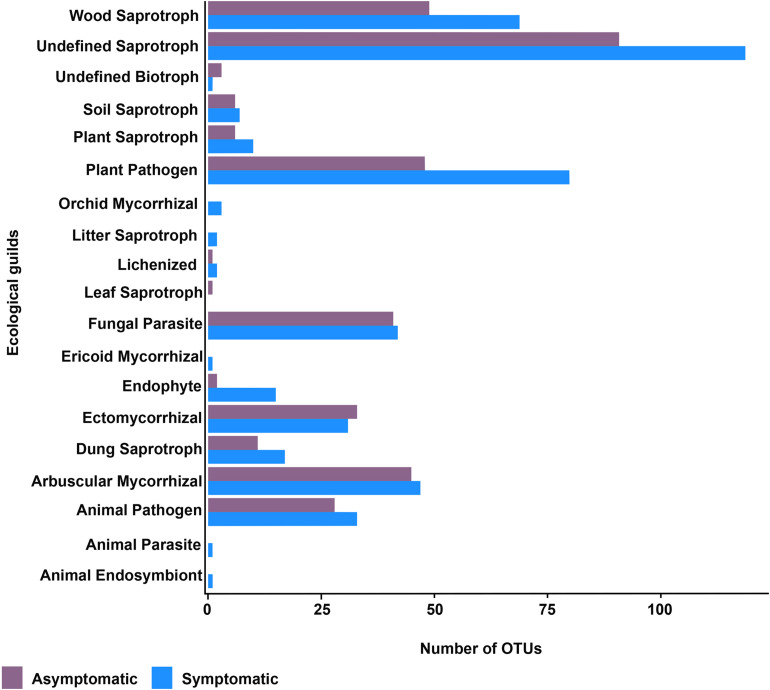
Ecological guilds of the rhizosphere fungal community of root rot asymptomatic and symptomatic avocado trees.

Fungal taxa were classified in different functional groups based on their uses of environmental resources with FUNGuild. All fungal OTUs were used in the predicted function analysis. Of the total 1,184 fungal OTUs, 539 (46%) were assigned to ecological categories, but only 391 (33%) OTUs had an assignment with probable and highly probable confidence ranks. The fungal trophic modes symbiotroph, saprotroph, and pathotroph seemed to be enriched in the rhizosphere of root rot symptomatic trees (Supplementary Figure S7). According to the ecological guilds, the saprotrophic fungi and plant pathogen fungi were represented by a larger number of OTUs in the rhizosphere of root rot symptomatic trees (Figure 5).

### Isolation and Pathogenicity Potential of Culturable Fungi Associated With Root Rot Symptomatic Trees

**FIGURE 6:**
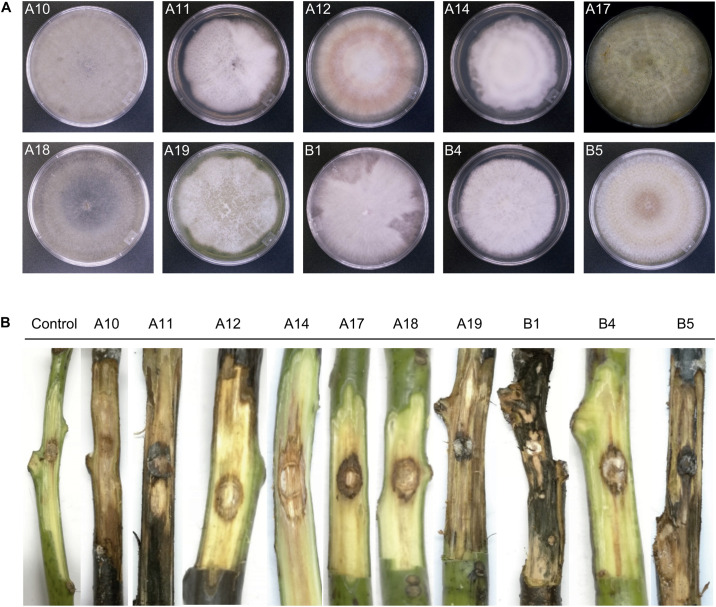
Morphology of fungal isolates from avocado root rot symptomatic trees **(A)** and representative necrotic lesions observed after 4 weeks in wound-inoculated stem segments **(B)**. The frontal side of colonies growing on PDA Petri dishes was visually registered **(A)** and the ID of each isolate is indicated. A10: *Lasiodiplodia pseudotheobromae*; A11: *Fusarium* sp.; A12: *Fusarium* sp.; A14: *Mortierella* sp.; A17: *F. equiseti*; A18: *Scytalidium* sp.; A19: *Fusarium* sp.; B1: *Fusarium* sp.; B4: *F. solani*; B5: *F. solani.* Frontal view of the internal lesions caused by each isolate **(B)** was also complemented with visual analysis of cross sections and the necrosis level is indicated in Table 2.

From 90 plates containing the three different culture media, 46 fungal isolates were recovered. These isolates were further classified into 10 morphotypes according to their morphological characteristics. Complementation with molecular identification based on the ITS1 and ITS4 sequences classified the isolates into four genera, being *Fusarium* the dominant genus with 7 isolates (A11, A12, A17, A19, B1, B4 and B5), 4 of them (A11, A12, B4 and B5) phylogenetically close to *F. solani* (Table 2). The additional genera represented by one single isolate were *Lasiodiplodia*, *Mortierella*, and *Scytalidium* (Table 2). The upper surface of the mycelial growth of fungal isolates was visually registered (Figure 6A).

**TABLE 2:** rDNA-ITS molecular identification of fungal isolates associated with root rot symptomatic avocado trees and necrosis severity on avocado stems as assessed in pathogenicity tests.

ID isolate	Taxonomic assignment	GenBank accession number	NCBI best match (accession number)	Identity (%)	Necrosis severity^*a*^
A10	*Lasiodiplodia pseudotheobromae*	MT571538	*Lasiodiplodia pseudotheobromae* (MN887212.1)	100	1
A11	*Fusarium* sp.	MT571539	*Fusarium* sp. (MH397492.1)	99.8	2
A12	*Fusarium* sp.	MT571540	*Fusarium* sp. (MH397492.1)	99.8	1
A14	*Mortierella* sp.	MT571541	*Mortierella* sp. (MT366005.1)	99.8	1
A17	*Fusarium equiseti*	MT571542	*Fusarium equiseti* (MG650603.1)	99.8	2
A18	*Scytalidium* sp.	MT571543	*Scytalidium* sp. (MH268087.1)	99.8	1
A19	*Fusarium* sp.	MT571544	*Fusarium* sp. (JF505287.1)	99.0	1
B1	*Fusarium* sp.	MT571545	*Fusarium* sp. (MH777054.1)	99.8	3
B4	*Fusarium solani*	MT571546	*Fusarium solani* (MN326475.1)	100	1
B5	*Fusarium solani*	MT571547	*Fusarium solani* (MN326475.1)	97.8	3

*^a^ Values assigned indicate the severity of the internal necrotic lesions observed in wound-inoculated stem segments of avocado after 4 weeks, following this classification: a value of 0 means no symptoms except discoloration at the wound site (the same as control); a value of 1 indicates moderate vascular damage (necrotic lesions surrounding the site of inoculation); a value of 2 refers to advanced damage (external necrosis, wilting and extended necrotic lesion through vascular tissue); a value of 3 indicates severe damage (generalized necrosis through the whole stem tissues).*

The functional prediction results obtained from the fungal community suggested that the pathotropy guild was enriched in the rhizosphere of symptomatic trees; we thus complemented the obtention of fungal isolates with the evaluation of their pathogenic potential, by testing their ability to necrotize vascular tissue of asymptomatic stems of c.v. Hass avocado. One representative isolate per fungal morphotype was tested (10 isolates). At 4 weeks after inoculation (wai), all the stems showed external necrotizing signs. Visual examination of internal damage corroborated the necrotic ability of all the screened isolates, as compared with mock inoculated stems (Figure 6B). The most severe damage was registered for isolates B1 and B5, tentatively identified as *Fusarium* sp. and *F. solani* respectively, which produced generalized necrosis and visible mycelial growth along and through the whole stem tissues (Figure 6B and Table 2). Isolates A11 and A17, tentatively identified as *Fusarium* sp. and *F. equiseti*, scored a value of 2 in the scale of necrosis severity (Figure 6B and Table 2). Although causing less generalized damage, the other tested isolates also showed an important necrotizing ability, scoring a value of 1 in the scale of necrosis severity (Figure 6B and Table 2).

## Discussion

We characterized the bacterial and fungal communities associated with the rhizosphere of root-rot asymptomatic and symptomatic avocado trees in an orchard located in the State of Veracruz, Mexico, and predicted their potential functions. Our results confirmed that root-rot infection modified the composition of microbial rhizosphere assemblages and the relative abundance of some bacterial and fungal taxa within the community, although, contrary to our hypothesis, no change in rhizosphere bacterial diversity was detected between root-rot asymptomatic and symptomatic avocado trees. Future research should aim at evaluating the impact of root rot on the avocado rhizosphere microbiome under controlled conditions, and control for factors such as time since infection or level of infection, in order to confirm the lack of influence of this disease on the rhizosphere microbial diversity.

The enrichment in specific taxonomic groups in the rhizosphere of symptomatic trees may be due to possible shifts in the ecological niche of the microbial community caused by root rot. Root necrosis induces the leakage of carbon-rich exudates, providing favorable niches for the proliferation of certain microbial populations due to their distinct substrate preferences (Zhalnina et al., 2018) or to their resistance to antimicrobial compounds released by the plant to inhibit the growth of the pathogen (Pascale et al., 2020). The higher relative abundance of Pseudomonadales, Burkholderiales and other Beta- and Gamma-Proteobacteria in the rhizosphere of root-rot symptomatic trees is consistent with this hypothesis, as these fast-growing bacteria are known for rapidly colonizing the rhizosphere in response to the liberation of labile sources of carbon in root exudates (Eilers et al., 2010; Trivedi et al., 2012). Moreover, these taxa have been associated with soil suppressiveness and plant protection against fungal diseases (Mendes et al., 2011), for example through the production of antifungal 2,4-diacetylphloroglucinol (DAPG; Bergsma-Vlami et al., 2005), the emission of antifungal and anti-oomycete volatile organic compounds such as dimethyl disulfide or 1-undecene (Hunziker et al., 2015; Báez-Vallejo et al., 2020) or through the production of siderophores (Paulsen et al., 2005). Furthermore, the genus *Pseudomonas* has been reported to be able to evade or reduce plant defenses in order to colonize the rhizosphere (Liu et al., 2018; Yu et al., 2019). It is also noteworthy that the bacterial taxa that were associated with the asymptomatic status in the present study, namely Acidobacteria, Actinobacteria and Firmicutes, were found to be indicators of the disease in the rhizosphere of Huanglongbing-affected citrus trees (Trivedi et al., 2012). Trivedi et al. (2012) attributed the enriched abundance of these taxa in the roots of diseased trees to their oligotrophic nature and their slow response to changes in root exudation patterns, which is likely why they were principally associated to intact roots in our study. Actinobacteria, *Bacillus* spp. and Rhizobiales, a bacterial taxon also found to be associated with the asymptomatic condition, are known to comprise plant growth promoting bacteria and have been reported to suppress or antagonize *P. cinnamomi* (You et al., 1996; Guevara-Avendaño et al., 2018; Vida et al., 2020); a decrease in their abundance may thus have further impact on the health of root-rot symptomatic trees.

Fungal taxa Leotiomycetes (in particular Helotiales) and Mortierellomycetes were amongst the most abundant fungi associated with the rhizosphere of root rot symptomatic avocado trees. Both taxa have been described as abundant in bulk and rhizosphere soils and as potentially important actors in the cycling of phosphorus (Tedersoo et al., 2009; Curlevski et al., 2010; Almario et al., 2017). The genus *Mortierella*, in particular, was reported by Shu et al. (2019) to be more abundant in the rhizosphere of root rot symptomatic avocado trees than in that of healthy trees in China, which was confirmed in our study. *Mortierella* spp. have been hypothesized to act as plant protective microbes, suppressing soil-borne pathogens through competition or enhanced plant nutrient uptake (Miao et al., 2016); however, a recent study from Mexico confirmed *Mortierella elongata* as an avocado pathogen, which suggests that *Mortierella* species may act as opportunistic phytopathogens in root-rot affected orchards (Hernández Pérez et al., 2018). Interestingly, isolate *Mortierella* sp. A14 was also retrieved in our culture-dependent approach as a fungal pathogen associated to root rot symptoms.

The predicted functional analysis of the rhizosphere bacterial communities showed that categories associated with the lipid and amino-acid metabolisms were promoted in the asymptomatic tree rhizospheres. To our knowledge, no previous reports have systematically characterized the lipid profile of avocado roots; however, evidence of highly expressed genes related to lipid metabolism have been found in healthy avocado roots (Ibarra-Laclette et al., 2015), which could explain the enrichment in the lipid metabolism category that was detected in the rhizosphere bacterial community of asymptomatic trees (Figure 4). On the other hand, the amino acid profile of avocado genotypes susceptible to root rot have been shown to be enriched in acidic and basic amino acids attractant to *P. cinnamomi* (Zentmyer, 1961; Botha and Kotzé, 1989); carbohydrates, however, were not a significant component of the chemoattractant root extracts (Botha and Kotzé, 1989). The incidence of root rot in the orchard sampled in this study corroborates the susceptibility of its avocado rootstock, thereby suggesting that an enrichment in amino acids could occur at the root level of these trees. Our results, nevertheless, contrasts with the results obtained by Shu et al. (2019), who suggested that the observed variations in microbial metabolism between root rot symptomatic and asymptomatic trees may affect the selection pressure that root rot causal agents exerted on the rhizosphere microbiome. These contrasting findings may due to the fact that our predictions of bacterial functions were based on 56% of the total OTUs detected in the rhizosphere bacterial community. Furthermore, future analyses with a functional genomic approach could clarify these opposite results in the predictive functions of the avocado rhizosphere microbiome.

Interestingly, sequences potentially related to chemotaxis and motility were more abundant in bacterial communities associated with the root rot symptomatic condition, which putatively emphasizes the importance of chemical signals for rhizosphere colonization by certain bacterial taxa. These predictions are consistent with the enrichment of *Pseudomonas* and *Burkholderia* species in the rhizosphere of root-rot symptomatic trees, as these Proteobacterial taxa are known producers of quorum sensing signals (Venturi and Keel, 2016). The categories of bacterial secretion system and two-component system also showed an increment in the bacterial community of symptomatic trees; these systems regulate important biological processes such as respiration, biofilm formation, motility, virulence, and antibiotic resistance (Tiwari et al., 2017; Schmidl et al., 2019). Fungal ecological guilds, assessed from 33% of fungal OTUs, were also marked by a clear difference between the asymptomatic and symptomatic avocado trees. As hypothesized, our results predict that the rhizosphere fungal community of root-rot symptomatic trees could be enriched in potential pathogens and saprotrophs, which is consistent with the larger proportion of Mortierellales, Pleosporales (Zhang et al., 2012) or Venturiales (Shen et al., 2020) detected in the rhizosphere of symptomatic trees. Opportunistic fungal pathogens may take advantage of root rot to colonize the roots of the susceptible host, whilst the release of root exudates following root rot is likely to attract saprotrophs to the rhizosphere. However, further studies should aim at confirming these findings with transcriptomic analyses to determine shifts in the functional diversity of the avocado rhizosphere microbiome due to root rot.

Culture-dependent assessment of fungi associated with root rot symptoms and subsequent pathogenicity tests allowed us to retrieve 10 fungal isolates with pathogenic activity on avocado tissues. These isolates mostly belonged to the genus *Fusarium*, which confirms the findings by Carranza-Rojas et al. (2015) who found several *Fusarium* species (Hypocreales), among which *F. solani* and *F. sambucinum*, to be associated with avocado root rot. Other fungal genera such as *Lasiodiplodia* (Botryosphaeriales), *Scytalidium* (Helotiales) and *Mortierella* (Mortierellales) have been previously reported as avocado pathogens in Peru, Spain, South Africa and Mexico (Alama et al., 2006; Coertzen and Fourie, 2017; Hernández Pérez et al., 2018; Arjona-Girona et al., 2019). Sequencing of the fungal microbiome confirmed that Helotiales and Mortierellales were dominant taxa in the rhizosphere of root rot symptomatic trees, which suggests that these taxa could act as opportunistic fungal pathogens following root rot infection. The potential pathogenicity of *Mortierella* sp., *Fusarium* spp., *Lasiodiplodia* sp. and *Scytalidium* sp., is reported for the first time for the State of Veracruz, Mexico, where avocado orchards are quickly expanding.

Plant microbiome studies offer a great area of opportunity to design innovative management strategies for the control of plant pathogens (Massart et al., 2015). Understanding the shifts in the avocado rhizosphere microbiome due to root rot could help developing diagnostic tools, including identifying microbial taxa associated to the disease causal agents, and direct the selection of plant growth-promoting bacteria that could be used in consortia for biofertilization. Further studies should be directed at elucidating the functional traits of microorganisms exclusively associated with the healthy or the sick conditions, as this could help us identify disease-suppressive microorganisms that could act as a first barrier against Phytophthora infection (Mendes et al., 2011; Trivedi et al., 2020). Such knowledge could be used as a basis to develop microbial consortia for the biocontrol of avocado root rot.

## Conclusion

The analysis of microbial communities in the avocado rhizosphere showed that root rot did not affect species richness, diversity or community structure. However, root rot induced changes in the proportion of several bacterial taxa within the community, increasing the relative abundance of Pseudomonadales and Burkholderiales whilst reducing that of Actinobacteria, *Bacillus* spp. and Rhizobiales. These shifts are likely to be mediated by the liberation of carbohydrates through necrotized roots and by changes in root exudate patterns as a response to pathogen infection. The rhizosphere of root rot symptomatic trees was also enriched in *Mortierella* spp., which are, together with *Fusarium* spp., *Lasiodiplodia* sp. and *Scytalidium* sp., probable opportunistic phytopathogens. Their potential pathogenicity in avocado is reported here for the first time for the State of Veracruz, Mexico. These results confirmed the predicted enrichment in potential fungal pathogens and saprotrophs in the rhizosphere of root-rot symptomatic trees, as determined by the analysis of fungal ecological guilds. Finally, root rot also potentially modified the predicted functions carried out by rhizobacteria, reducing the number of sequences related to the lipid and amino-acid metabolism categories whilst tentatively promoting categories associated with quorum sensing, virulence, and antibiotic resistance. Altogether, these results show the potential of the plant microbiome for the development of diagnostic and disease management tools, and call for a better understanding of the shifts in rhizosphere microbial functional traits due to root rot, as these are key to develop microbial consortia for the biocontrol of the disease.

## Data Availability Statement

The datasets presented in this study can be found in online repositories. The names of the repository/repositories and accession number(s) can be found in the article/Supplementary Material.

## Author Contributions

IS-G conducted the study, analyzed the metagenomic data, and wrote the manuscript. OC-L, EC-M, and EG-A harvested the samples, extracted the DNA and analyzed the culturable fungi data. DD analyzed the metagenomic data. EG-S supervised the identification of fungal isolates. VP-C designed and supervised the sequencing library construction. FR and AM-B designed and supervised the study, wrote the manuscript, and provided the funding. All authors revised the manuscript and contributed to its final version.

## Conflict of Interest

The authors declare that the research was conducted in the absence of any commercial or financial relationships that could be construed as a potential conflict of interest.

## Footnotes

1 www.ncbi.nlm.nih.gov

2 https://unite.ut.ee/

## Supplementary Material

Supplementary Figure 1 Rarefaction curves of the number of observed OTUs of the bacterial **(A)** and fungal **(B)** community associated with root rot asymptomatic and symptomatic avocado trees.

Supplementary Figure 2 Non-metric multidimensional scaling (NMDS) plot based on UniFrac unweighted distance of the bacterial community structure associated with the roots of root rot asymptomatic and symptomatic avocado trees (stress value = 0.15).

Supplementary Figure 3 Taxonomic composition of the rhizosphere bacterial community at the level of class **(A)** and order **(B)** associated with root rot asymptomatic and symptomatic avocado trees. Low abundance taxonomic groups (relative abundance < 1%) were reported as Others. NA means not assigned. The asterisk indicates significant difference in taxa relative abundance between root rot asymptomatic and symptomatic trees (For bacteria: *P* < 0.05, Mann-Whitney-Wilcoxon test; for fungi: log2fdc ≠ 0 and Gfold(0.01) ≠ 0, Gfold algorithm).

Supplementary Figure 4 Taxonomic composition of the rhizosphere fungal community at the level of phylum **(A)** and order **(B)** of root rot asymptomatic and symptomatic avocado trees. Low abundance taxonomic groups (relative abundance < 1%) were reported as Others. NA means not assigned. The asterisk indicates significant difference in taxa relative abundance between root rot asymptomatic and symptomatic trees (For bacteria: *P* < 0.05, Mann-Whitney-Wilcoxon test; for fungi: log2fdc ≠ 0 and Gfold(0.01) ≠ 0, Gfold algorithm).

Supplementary Figure 5 Difference in the mean abundance of each categorized gene function, at level 1 KEGG orthology (KO), of the rhizosphere bacterial community of root rot asymptomatic and symptomatic trees. The *q*-values were derived from a White’s non-parametric *t*-test with Benjamini-Hochberg correction.

Supplementary Figure 6 Differential functions of the avocado rhizosphere bacterial community between root rot asymptomatic and symptomatic trees. The sequences were associated to bacterial chemotaxis **(A)**, bacterial motility proteins **(B)**, bacterial secretion system **(C)** and two- component system **(D)**. The *q*-values were derived from a White’s non-parametric *t*-test with Benjamini-Hochberg correction.

Supplementary Figure 7 Fungal trophic modes detected in the rhizosphere of root rot asymptomatic and symptomatic trees. The trophic mode was assigned using the FUNGuild database and based on 33% of fungal OTUs (OTUs with probable and highly probable confidence ranks).
