# Supplementary figures and images for: Phytophthora Root Rot Modifies the Composition of the Avocado Rhizosphere Microbiome and Increases the Abundance of Opportunistic Fungal Pathogens
Source: Front Microbiol. 2021 Jan 12;11:574110. doi: 10.3389/fmicb.2020.574110 (PMC7835518; doi:10.3389/fmicb.2020.574110)

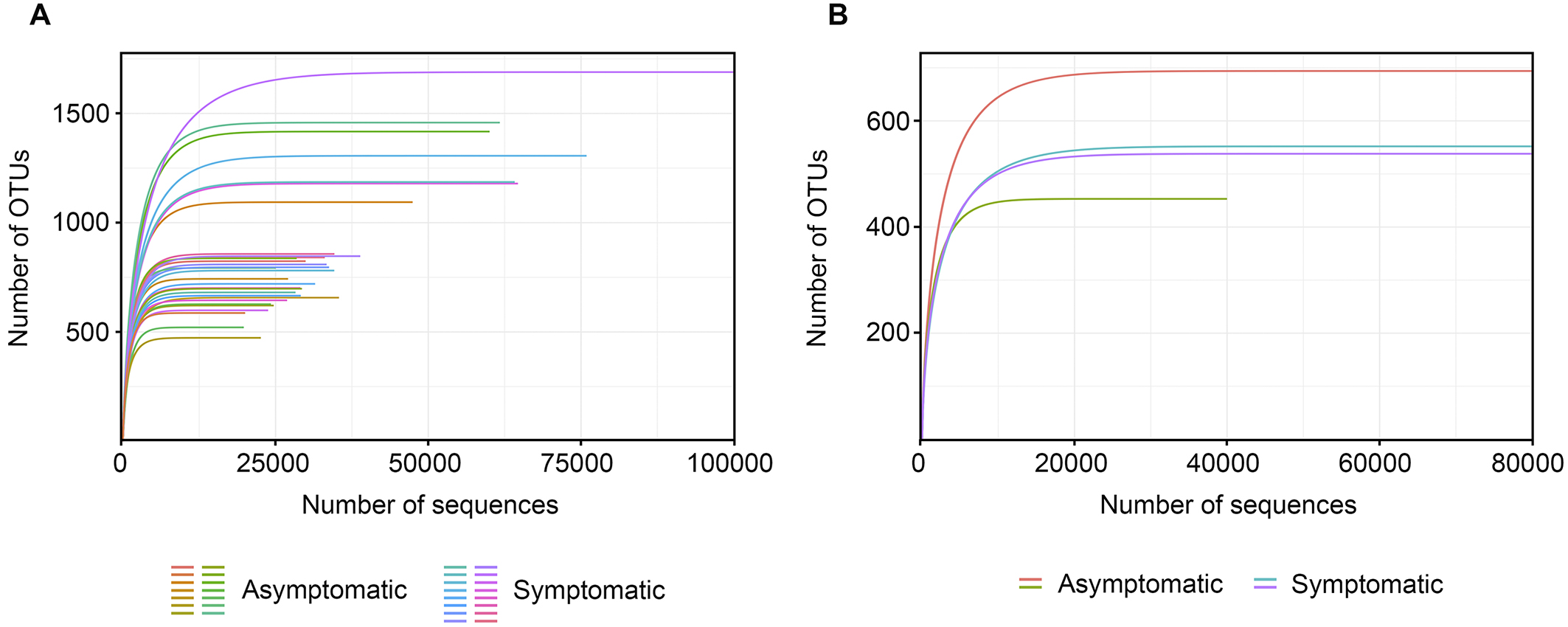

Supplement: Supplementary Figure 1 — Rarefaction curves of the number of observed OTUs of the bacterial (A) and fungal (B) community associated with root rot asymptomatic and symptomatic avocado trees. [file Image_1.jpg]

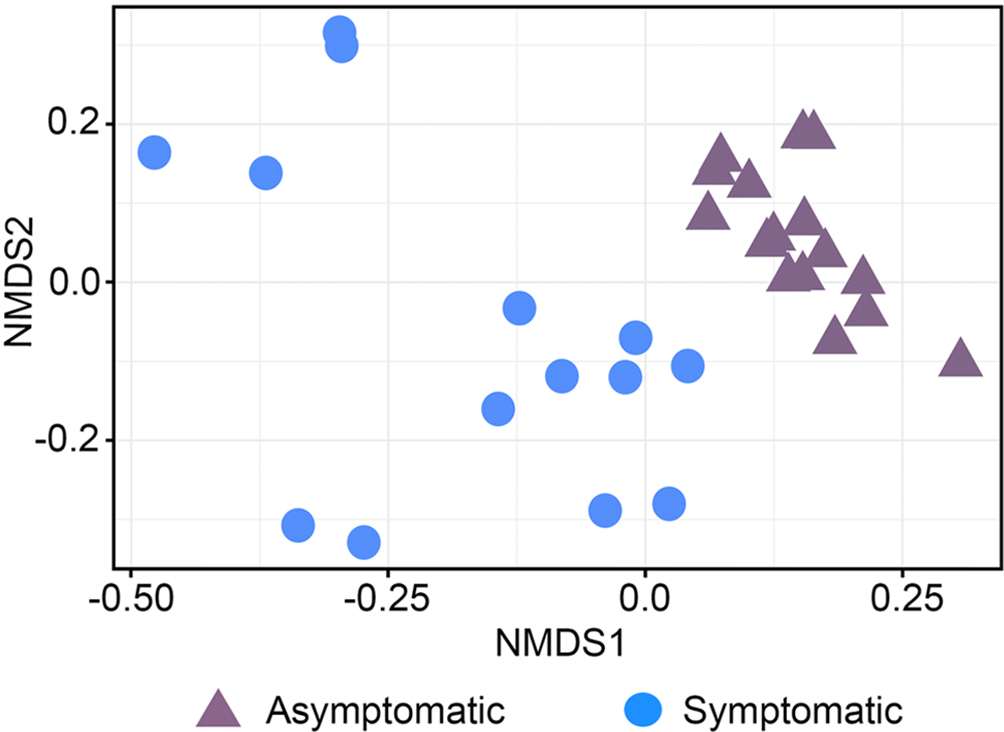

Supplement: Supplementary Figure 2 — Non-metric multidimensional scaling (NMDS) plot based on UniFrac unweighted distance of the bacterial community structure associated with the roots of root rot asymptomatic and symptomatic avocado trees (stress value = 0.15). [file Image_2.jpg]

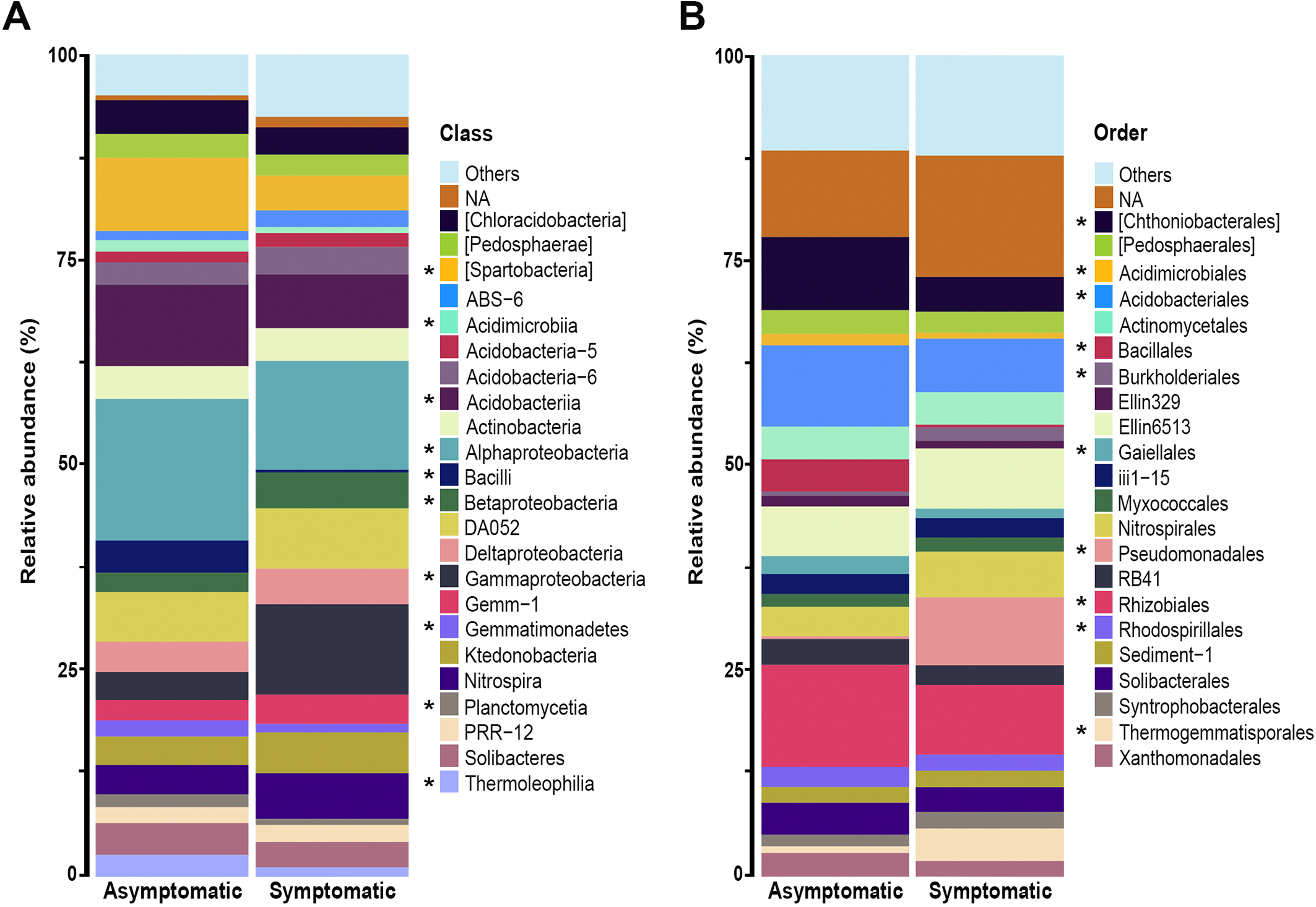

Supplement: Supplementary Figure 3 — Taxonomic composition of the rhizosphere bacterial community at the level of class (A) and order (B) associated with root rot asymptomatic and symptomatic avocado trees. Low abundance taxonomic groups (relative abundance < 1%) were reported as Others. NA means not assigned. The asterisk indicates significant difference in taxa relative abundance between root rot asymptomatic and symptomatic trees (For bacteria: P < 0.05, Mann-Whitney-Wilcoxon test; for fungi: log2fdc ≠ 0 and Gfold(0.01) ≠ 0, Gfold algorithm). [file Image_3.jpg]

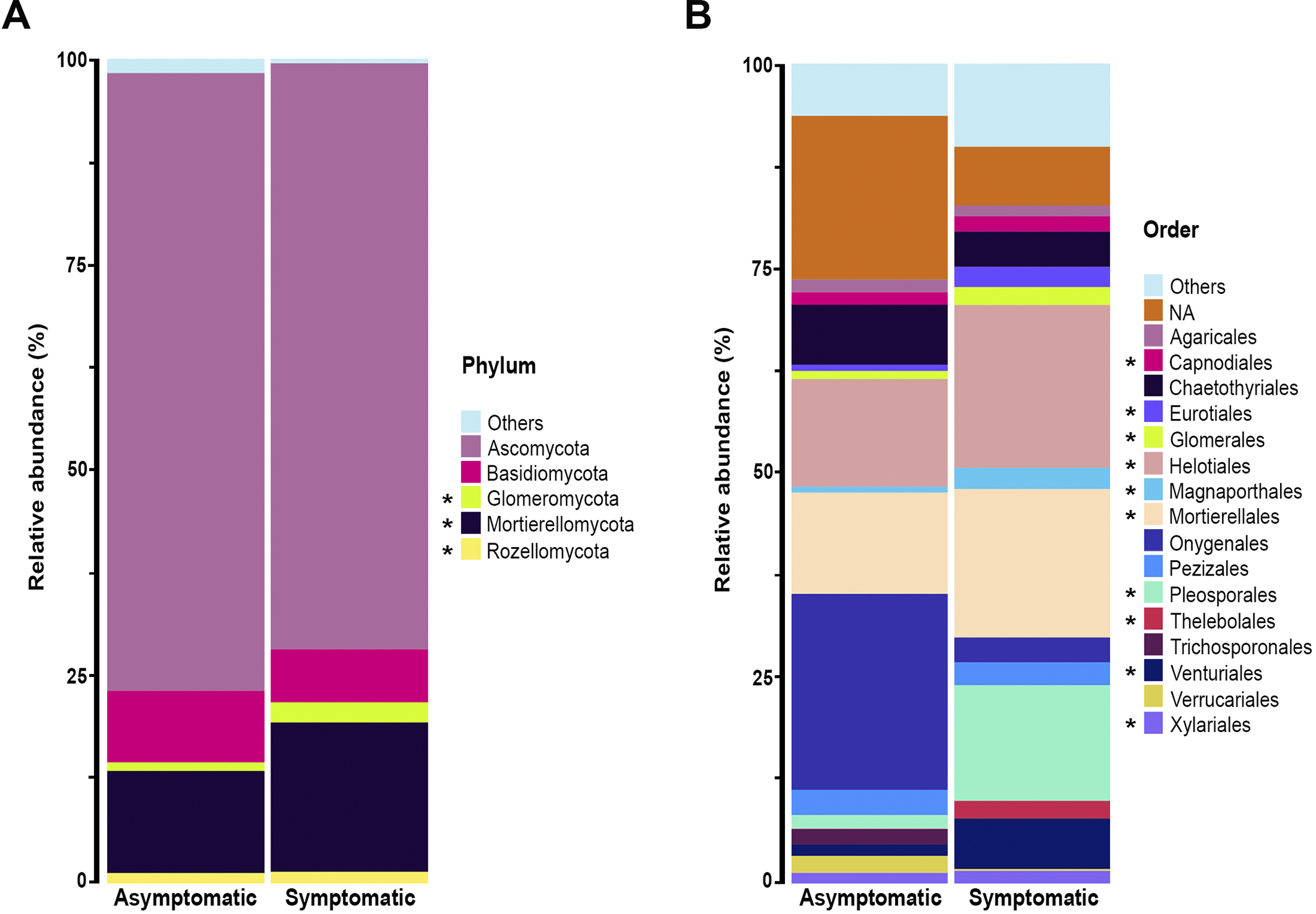

Supplement: Supplementary Figure 4 — Taxonomic composition of the rhizosphere fungal community at the level of phylum (A) and order (B) of root rot asymptomatic and symptomatic avocado trees. Low abundance taxonomic groups (relative abundance < 1%) were reported as Others. NA means not assigned. The asterisk indicates significant difference in taxa relative abundance between root rot asymptomatic and symptomatic trees (For bacteria: P < 0.05, Mann-Whitney-Wilcoxon test; for fungi: log2fdc ≠ 0 and Gfold(0.01) ≠ 0, Gfold algorithm). [file Image_4.jpg]

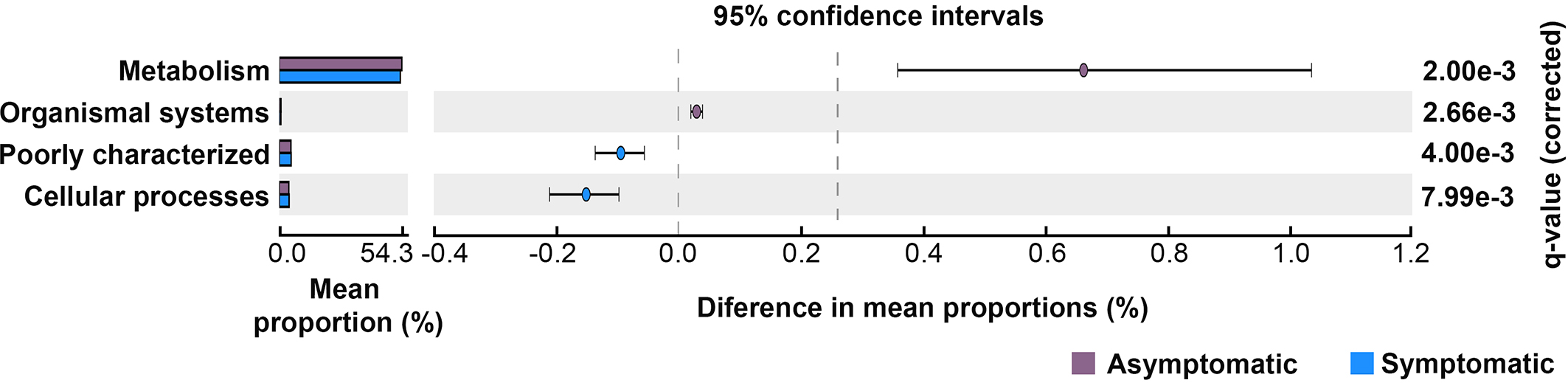

Supplement: Supplementary Figure 5 — Difference in the mean abundance of each categorized gene function, at level 1 KEGG orthology (KO), of the rhizosphere bacterial community of root rot asymptomatic and symptomatic trees. The q-values were derived from a White’s non-parametric t-test with Benjamini-Hochberg correction. [file Image_5.jpg]

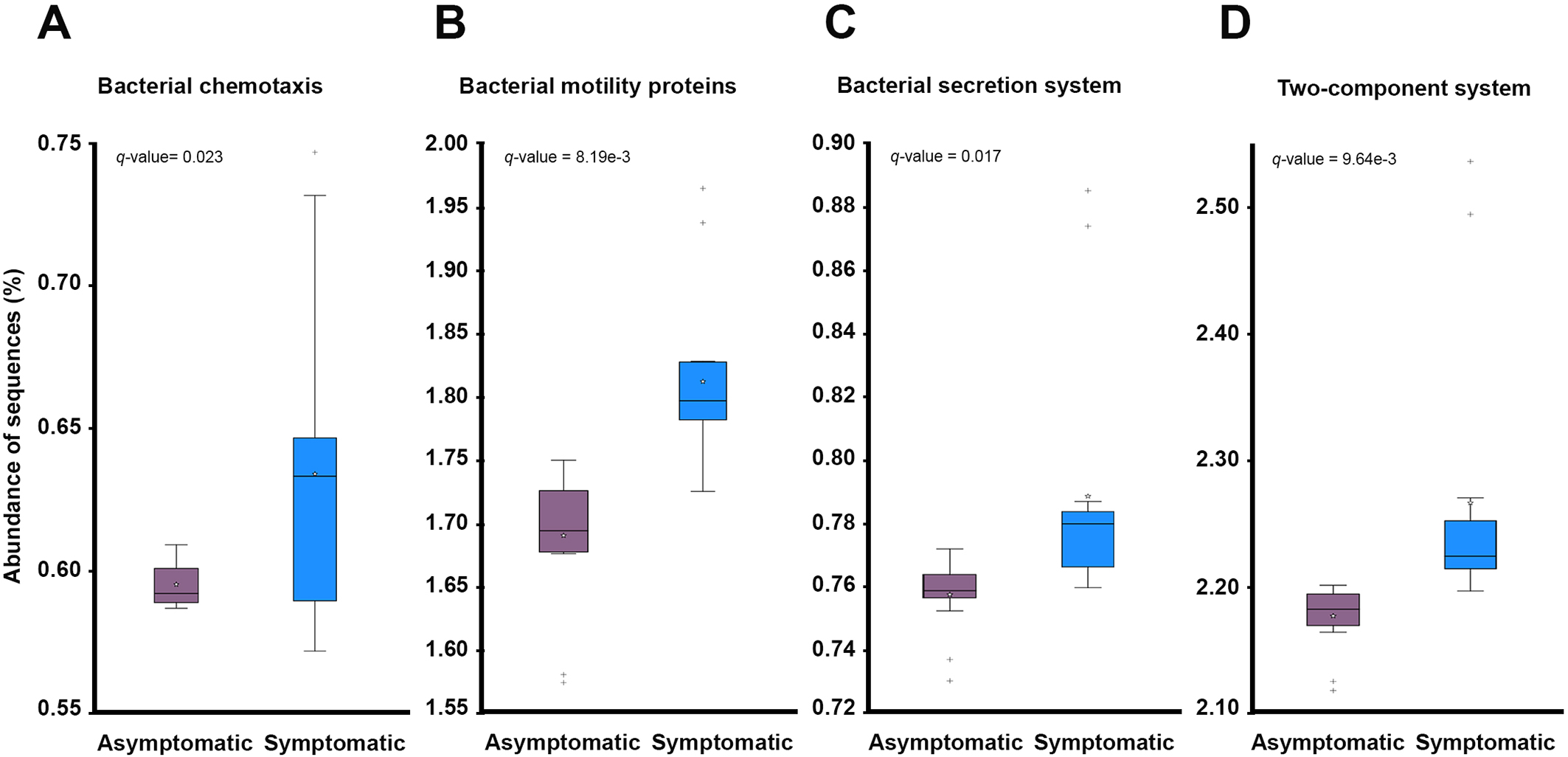

Supplement: Supplementary Figure 6 — Differential functions of the avocado rhizosphere bacterial community between root rot asymptomatic and symptomatic trees. The sequences were associated to bacterial chemotaxis (A), bacterial motility proteins (B), bacterial secretion system (C) and two- component system (D). The q-values were derived from a White’s non-parametric t-test with Benjamini-Hochberg correction. [file Image_6.jpg]

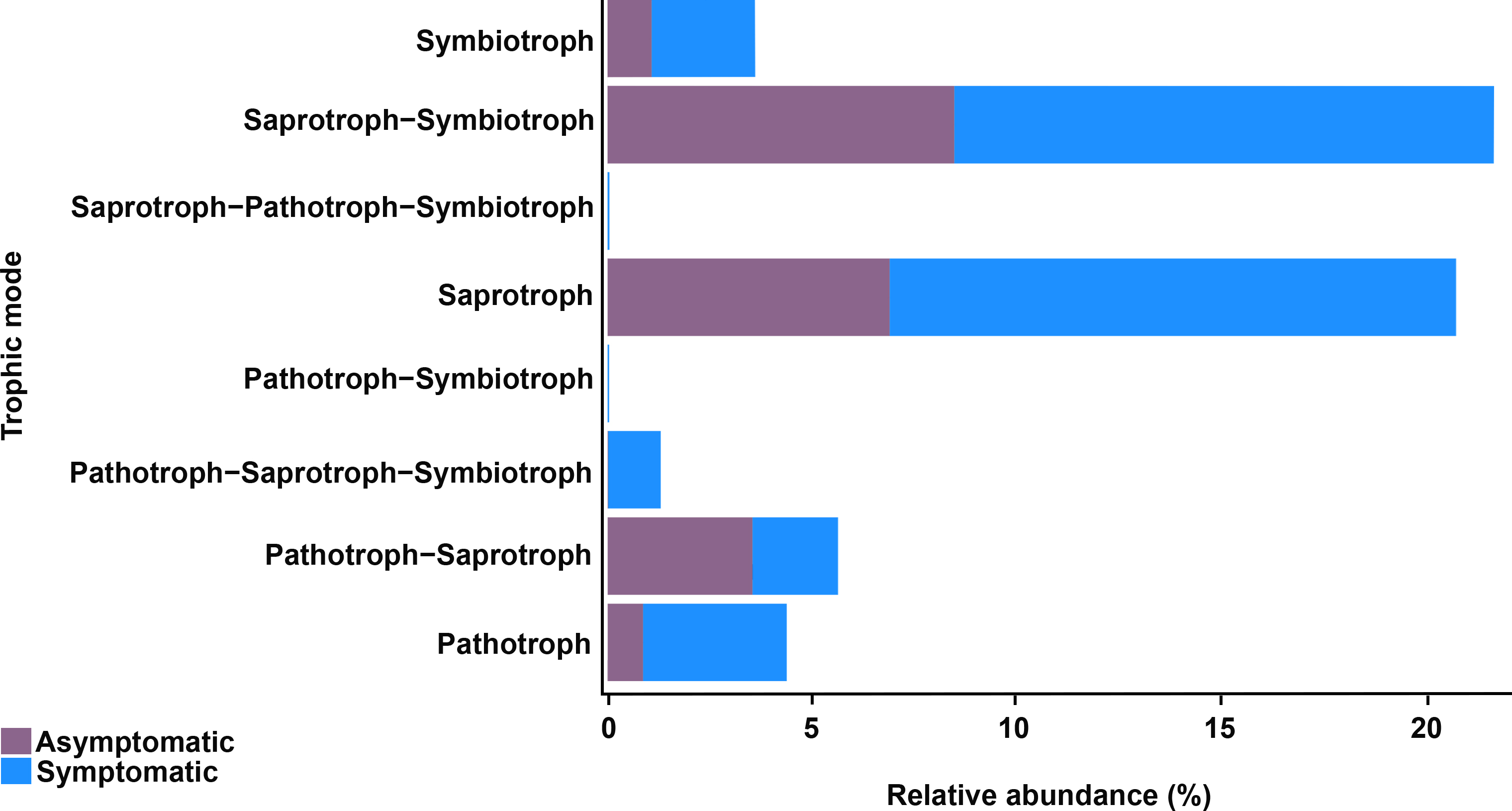

Supplement: Supplementary Figure 7 — Fungal trophic modes detected in the rhizosphere of root rot asymptomatic and symptomatic trees. The trophic mode was assigned using the FUNGuild database and based on 33% of fungal OTUs (OTUs with probable and highly probable confidence ranks). [file Image_7.jpg]
