# Supplementary material for: Phytophthora Root Rot Modifies the Composition of the Avocado Rhizosphere Microbiome and Increases the Abundance of Opportunistic Fungal Pathogens
Source: Front Microbiol. 2021 Jan 12;11:574110. doi: 10.3389/fmicb.2020.574110 (PMC7835518; doi:10.3389/fmicb.2020.574110)
Supplement: Supplementary file 8 [file Table_1.docx]

Supplementary Material

**TABLE S1** Summary of sequencing data processing from rhizosphere soil samples of root rot asymptomatic and symptomatic avocado trees

|  | **Raw sequences** | **High-quality sequences** | **Average sequence length (pb)** | **OTUs** |
| --- | --- | --- | --- | --- |
| **Bacteria** |  |  |  |  |
| Asymptomatic | 1,457,094 | 707,549 | 267 | 3,424 |
| Symptomatic | 1,100,460 | 455,615 | 267 |  |
| **Fungi** |  |  |  |  |
| Asymptomatic^*^ | 1,982,786 | 123,267 | 131 | 1,184 |
| Symptomatic^§^ | 2,203,106 | 170,232 | 131 |  |

^*^ Proportion of merged paired-end reads: 4.36%

^§^ Proportion of merged paired-end reads: 5.43%
