# Supplementary material for: Phytophthora Root Rot Modifies the Composition of the Avocado Rhizosphere Microbiome and Increases the Abundance of Opportunistic Fungal Pathogens
Source: Front Microbiol. 2021 Jan 12;11:574110. doi: 10.3389/fmicb.2020.574110 (PMC7835518; doi:10.3389/fmicb.2020.574110)
Supplement: Supplementary file 9 [file Table_2.docx]

Supplementary Material

**TABLE S2** Permutational multivariate analysis of variance (PERMANOVA) based on UniFrac weighted and unweighted distances of the bacterial community structure in the rhizosphere of root rot asymptomatic and symptomatic avocado trees

| **Distance** | **df** | **SS** | **MS** | **F** | **R^2^** | **P** |
| --- | --- | --- | --- | --- | --- | --- |
| Weighted UniFrac | 1 | 0.0009631 | 0.0009631 | 1.0981 | 0.03774 | 0.329 |
| Unweighted UniFrac | 1 | 0.2255 | 0.2255 | 1.1126 | 0.03822 | 0.305 |
