# Supplementary material for: Phytophthora Root Rot Modifies the Composition of the Avocado Rhizosphere Microbiome and Increases the Abundance of Opportunistic Fungal Pathogens
Source: Front Microbiol. 2021 Jan 12;11:574110. doi: 10.3389/fmicb.2020.574110 (PMC7835518; doi:10.3389/fmicb.2020.574110)
Supplement: Supplementary file 10 [file Table_3.docx]

Supplementary Material

**TABLE S3** Taxonomic composition, at the phylum level*,* of the rhizosphere bacterial community of root rot asymptomatic and symptomatic avocado trees

|  | **Relative abundance (%)** | | **Wilcoxon rank sum test** | |
| --- | --- | --- | --- | --- |
| ***Phylum*** | **Asymptomatic** | **Symptomatic** | **p-value** | **p-adjusted FDR** |
| Proteobacteria | 26.7948934 | 32.9745509 | 0.02348 | 0.05674333 |
| Acidobacteria | 28.7673363 | 27.0061346 | 0.7238 | 0.839608 |
| Verrucomicrobia | 12.0969714 | 7.1885254 | 6.03E-05 | 0.00079267 |
| Actinobacteria | 8.2090428 | 6.1150313 | 0.005016 | 0.02078057 |
| Chloroflexi | 5.1350507 | 7.0098658 | 0.4176 | 0.57668571 |
| Nitrospirae | 3.5779854 | 5.5571041 | 0.01884 | 0.04966909 |
| Gemmatimonadetes | 4.4483138 | 4.631542 | 0.8516 | 0.91468148 |
| Firmicutes | 3.9054539 | 0.326811 | 3.57E-06 | 0.00010353 |
| Planctomycetes | 2.5104975 | 1.6325187 | 0.006472 | 0.023461 |
| WS3 | 1.9341417 | 2.0635844 | 0.6929 | 0.83725417 |
| AD3 | 1.3505778 | 2.255413 | 0.3286 | 0.47647 |
| Bacteroidetes | 0.5357933 | 1.7958144 | 0.001472 | 0.0085376 |
| **Others** | **Relative abundance (< 1%)** | |  |  |
| Chlorobi | 0.0565332 | 0.2805 | 0.008936 | 0.02879378 |
| GAL15 | 0.0572399 | 0.2787441 | 0.07661 | 0.15869214 |
| OD1 | 0.1085437 | 0.167466 | 0.1499 | 0.2896375 |
| Elusimicrobia | 0.1009117 | 0.1044742 | 0.6305 | 0.79497826 |
| TM7 | 0.1471276 | 0.0572852 | 0.004452 | 0.02078057 |
| WPS-2 | 0.0777331 | 0.1086444 | 0.8323 | 0.91468148 |
| Armatimonadetes | 0.0204933 | 0.1257641 | 0.0112 | 0.03248 |
| GN04 | 0 | 0.1358603 | 0.0002465 | 0.00178713 |
| FCPU426 | 0.0489012 | 0.0733075 | 0.1598 | 0.2896375 |
| Chlamydiae | 0.0378772 | 0.0190951 | 0.239 | 0.36478947 |
| TM6 | 0.0418346 | 0.0149249 | 0.9425 | 0.9526 |
| Fibrobacteres | 0 | 0.0427993 | 8.20E-05 | 0.00079267 |
| Cyanobacteria | 0.0216239 | 0.0158028 | 0.5554 | 0.73211818 |
| Spirochaetes | 0.00806 | 0.00812 | 0.9526 | 0.9526 |
| OP3 | 0 | 0.0103157 | 0.06066 | 0.13531846 |
| GN02 | 0.00382 | 0 | 0.1945 | 0.31336111 |
| Tenericutes | 0.00325 | 0 | 0.1945 | 0.31336111 |
