# Supplementary material for: Phytophthora Root Rot Modifies the Composition of the Avocado Rhizosphere Microbiome and Increases the Abundance of Opportunistic Fungal Pathogens
Source: Front Microbiol. 2021 Jan 12;11:574110. doi: 10.3389/fmicb.2020.574110 (PMC7835518; doi:10.3389/fmicb.2020.574110)
Supplement: Supplementary file 11 [file Table_4.docx]

Supplementary Material

**TABLE S4** Taxonomic composition, at the class level, of the rhizosphere bacterial community of root rot asymptomatic and symptomatic avocado trees

|  | **Relative abundance (%)** | | **Wilcoxon rank sum test** | |
| --- | --- | --- | --- | --- |
| **Class** | **Asymptomatic** | **Symptomatic** | **p-value** | **p-adjusted FDR** |
| Alphaproteobacteria | 17.21477947 | 13.23376096 | 0.0000719 | 0.00091225 |
| Acidobacteria | 9.908854369 | 6.521514875 | 0.008297 | 0.02953732 |
| Gammaproteobacteria | 3.410788511 | 10.98010382 | 0.004405 | 0.01866881 |
| DA052 | 6.050181684 | 7.330311776 | 1 | 1 |
| [Spartobacteria] | 8.904400967 | 4.238227451 | 0.0000204 | 0.00052332 |
| Nitrospira | 3.577985412 | 5.557104134 | 0.01884 | 0.05781931 |
| Ktedonobacteria | 3.462233711 | 4.954621775 | 0.9172 | 0.97449885 |
| Deltaproteobacteria | 3.672537167 | 4.307145287 | 0.2201 | 0.33201525 |
| Actinobacteria | 3.975696383 | 3.955532632 | 0.7552 | 0.86170256 |
| [Chloracidobacteria] | 4.094840075 | 3.325175861 | 0.4176 | 0.54656471 |
| Solibacteres | 3.888917941 | 3.081110148 | 0.07056 | 0.14272364 |
| Betaproteobacteria | 2.331711302 | 4.351919932 | 0.0001685 | 0.00166628 |
| Acidobacteria-6 | 2.699177018 | 3.339442292 | 0.1095 | 0.19888776 |
| Gemm-1 | 2.4740336 | 3.558267397 | 0.05856 | 0.12555209 |
| [Pedosphaerae] | 2.878669887 | 2.534815579 | 0.2048 | 0.32086842 |
| Bacilli | 3.893864595 | 0.326811014 | 0.00000357 | 0.00031773 |
| PRR-12 | 1.934141664 | 2.063584386 | 0.6929 | 0.81142237 |
| Thermoleophilia | 2.686174385 | 1.236131383 | 0.000014 | 0.00052332 |
| ABS-6 | 1.107202469 | 2.023199412 | 0.3085 | 0.44311212 |
| Acidobacteria-5 | 1.307753951 | 1.700339102 | 0.04378 | 0.10530865 |
| Gemmatimonadetes | 1.954634944 | 1.026305104 | 0.0000294 | 0.00052332 |
| Planctomycetia | 1.529788043 | 0.716613808 | 0.0000294 | 0.00052332 |
| Acidimicrobiia | 1.385204417 | 0.714418972 | 0.0000719 | 0.00091225 |
| Not assigned | 0.612395749 | 1.271907202 | 0.002579 | 0.01198385 |
| **Others** | **Relative abundance (< 1%)** | |  |  |
| Fibrobacteria | 0 | 0.042799293 | 0.000082 | 0.00091225 |
| Sphingobacteriia | 0.045367883 | 0.294766415 | 0.000293 | 0.0026077 |
| PAUC37f | 0.075895804 | 0.331639652 | 0.000379 | 0.00306646 |
| OPB56 | 0.020775946 | 0 | 0.0005751 | 0.00426533 |
| Flavobacteriia | 0.005794652 | 0.29015726 | 0.0007319 | 0.0050107 |
| Anaerolineae | 0.122677016 | 0.497130253 | 0.0011 | 0.00652667 |
| Opitutae | 0.050314537 | 0.166588018 | 0.001053 | 0.00652667 |
| SC3 | 0.025015935 | 0 | 0.001353 | 0.00752606 |
| Rubrobacteria | 0 | 0.021289905 | 0.00186 | 0.00973765 |
| RB25 | 0.038301234 | 0.134543419 | 0.002637 | 0.01198385 |
| BSV26 | 0.013002633 | 0.241651394 | 0.002693 | 0.01198385 |
| MSB-5A5 | 0 | 0.065186616 | 0.004718 | 0.01908646 |
| S085 | 0.125221009 | 0.299156086 | 0.006884 | 0.02663809 |
| Gemm-2 | 0.004663988 | 0.035995303 | 0.007465 | 0.02768271 |
| [Fimbriimonadia] | 0.02049328 | 0.125764077 | 0.0112 | 0.03753893 |
| Cytophagia | 0.161543582 | 0.625528132 | 0.01181 | 0.03753893 |
| GN15 | 0 | 0.046091547 | 0.01144 | 0.038 |
| Sva0725 | 0.032647916 | 0.0160223 | 0.01967 | 0.05835433 |
| TM7-1 | 0.076602469 | 0.045872063 | 0.02258 | 0.06482645 |
| Oscillatoriophycideae | 0 | 0.013169013 | 0.02671 | 0.07117382 |
| Clostridia | 0.011589303 | 0 | 0.02719 | 0.07117382 |
| Pla4 | 0.169882227 | 0.342174863 | 0.02615 | 0.07117382 |
| OM190 | 0.096247751 | 0.058382626 | 0.03538 | 0.08996629 |
| S035 | 0.06812249 | 0.014924882 | 0.04088 | 0.10106444 |
| TM1 | 0.019927948 | 0.166368535 | 0.04814 | 0.10985795 |
| [Methylacidiphilae] | 0.218924767 | 0.140249992 | 0.04806 | 0.10985795 |
| [Saprospirae] | 0.323087164 | 0.544099733 | 0.05856 | 0.12555209 |
| 5bav_B12 | 0 | 0.008559859 | 0.06066 | 0.12555209 |
| PBS-25 | 0 | 0.007681924 | 0.06066 | 0.12555209 |
| BPC102 | 0.003391991 | 0.014924882 | 0.08976 | 0.17752533 |
| Gemm-5 | 0.014981295 | 0.007681924 | 0.09565 | 0.18506196 |
| ML635J-21 | 0.005794652 | 0 | 0.1017 | 0.18856875 |
| C6 | 0.005794652 | 0 | 0.1017 | 0.18856875 |
| ZB2 | 0.052717197 | 0.078794596 | 0.1366 | 0.243148 |
| P2-11E | 0.194898163 | 0.16790492 | 0.1765 | 0.3080098 |
| Thermomicrobia | 0.003109325 | 0 | 0.1945 | 0.31473636 |
| 4C0d-2 | 0.015829292 | 0 | 0.1945 | 0.31473636 |
| GKS2-174 | 0.00381599 | 0 | 0.1945 | 0.31473636 |
| Mollicutes | 0.003250658 | 0 | 0.1945 | 0.31473636 |
| TK17 | 0.001554663 | 0.012071596 | 0.2055 | 0.32086842 |
| EC1113 | 0.029255924 | 0.057724175 | 0.2139 | 0.32822586 |
| Chlamydiia | 0.037877235 | 0.019095069 | 0.239 | 0.35451667 |
| Nostocophycideae | 0 | 0.002633803 | 0.3162 | 0.44311212 |
| Mb-NB09 | 0.025581267 | 0.014046948 | 0.3265 | 0.44311212 |
| koll11 | 0 | 0.002633803 | 0.3162 | 0.44311212 |
| Phycisphaerae | 0.691542211 | 0.504153726 | 0.3286 | 0.44311212 |
| TM7-3 | 0.023885272 | 0.002633803 | 0.3247 | 0.44311212 |
| 028H05-P-BN-P5 | 0.002685326 | 0 | 0.3851 | 0.51155075 |
| C0119 | 0.006925315 | 0.005267605 | 0.4391 | 0.56637536 |
| TK10 | 0.665960944 | 0.620918978 | 0.5194 | 0.66038 |
| Verrucomicrobiae | 0.044661218 | 0.036873237 | 0.5475 | 0.68630282 |
| Chloroflexi | 0.046781212 | 0.060796945 | 0.5657 | 0.69773562 |
| Elusimicrobia | 0.100911739 | 0.099865018 | 0.5723 | 0.69773562 |
| SJA-28 | 0.009893308 | 0.010535211 | 0.6692 | 0.79411733 |
| Gitt-GS-136 | 0.019079951 | 0.037312204 | 0.6682 | 0.79411733 |
| iii1-8 | 0.446753511 | 0.496252318 | 0.7552 | 0.86170256 |
| SM2F11 | 0.015263961 | 0.023045773 | 0.778 | 0.87648101 |
| JG37-AG-4 | 0.24337537 | 0.224312193 | 0.9502 | 0.97449885 |
| MB-A2-108 | 0.161967581 | 0.1815129 | 0.8843 | 0.97449885 |
| Ellin6529 | 0.366193719 | 0.33646829 | 0.9172 | 0.97449885 |
| Pla3 | 0.003674657 | 0.003292253 | 0.9234 | 0.97449885 |
| vadinHA49 | 0.010882639 | 0.007901408 | 0.933 | 0.97449885 |
| Spirochaetes | 0.008055979 | 0.008120892 | 0.9526 | 0.97449885 |
| SJA-4 | 0.041834558 | 0.014924882 | 0.9425 | 0.97449885 |
| ABY1 | 0.009751975 | 0.024143191 | 0.9776 | 0.98870909 |
