# Supplementary material for: Phytophthora Root Rot Modifies the Composition of the Avocado Rhizosphere Microbiome and Increases the Abundance of Opportunistic Fungal Pathogens
Source: Front Microbiol. 2021 Jan 12;11:574110. doi: 10.3389/fmicb.2020.574110 (PMC7835518; doi:10.3389/fmicb.2020.574110)
Supplement: Supplementary file 12 [file Table_5.docx]

Supplementary Material

**TABLE S5** Taxonomic composition, al the order level, of the rhizosphere bacterial community of PRR asymptomatic and symptomatic avocado trees

|  | **Relative abundance (%)** | | **Wilcoxon rank sum test** | |
| --- | --- | --- | --- | --- |
| **Order** | **Asymptomatic** | **Symptomatic** | **p-value** | **p-adjusted FDR** |
| Rhizobiales | 12.4425305 | 8.4727237 | 0.0000603 | 0.000919575 |
| Acidobacteriales | 9.9088544 | 6.5215149 | 0.008297 | 0.031632313 |
| Ellin6513 | 6.0501817 | 7.3303118 | 1 | 1 |
| [Chthoniobacterales] | 8.904401 | 4.2382275 | 0.0000204 | 0.000355543 |
| Nitrospirales | 3.5779854 | 5.5571041 | 0.01884 | 0.056060488 |
| Pseudomonadales | 0.3541804 | 8.2571908 | 0.0000157 | 0.000355543 |
| Actinomycetales | 3.9756964 | 3.9555326 | 0.7552 | 0.837585455 |
| Solibacterales | 3.8863739 | 3.0236055 | 0.05856 | 0.123177931 |
| RB41 | 3.1213386 | 2.3873226 | 0.2361 | 0.331082759 |
| [Pedosphaerales] | 2.8786699 | 2.5348156 | 0.2048 | 0.302060241 |
| Xanthomonadales | 2.9050992 | 2.0466842 | 0.03961 | 0.092931154 |
| iii1-15 | 2.433895 | 2.3754705 | 0.8843 | 0.94635614 |
| Thermogemmatisporales | 0.8178939 | 3.9432416 | 0.001698 | 0.009006783 |
| Rhodospirillales | 2.438559 | 1.9670116 | 0.003863 | 0.016251241 |
| Bacillales | 3.8938646 | 0.326811 | 0.00000357 | 0.00014518 |
| Sediment-1 | 1.9020591 | 1.9766689 | 0.8516 | 0.927635714 |
| Syntrophobacterales | 1.413895 | 2.0093719 | 0.1515 | 0.260323944 |
| Gaiellales | 2.1635251 | 1.1270481 | 0.0000855 | 0.000948273 |
| Myxococcales | 1.5614466 | 1.7115328 | 1 | 1 |
| Ellin329 | 1.30097 | 0.9123931 | 0.04831 | 0.111204151 |
| Burkholderiales | 0.5244866 | 1.6327382 | 0.0000204 | 0.000355543 |
| Acidimicrobiales | 1.3852044 | 0.714419 | 0.0000719 | 0.000948273 |
| Not assigned | 10.5270448 | 14.7530261 | 0.0001204 | 0.001224067 |
| **Others** | **Relative abundance (< 1%)** | |  |  |
| Solirubrobacterales | 0.5226493 | 0.1090833 | 0.00000353 | 0.00014518 |
| Planctomycetales | 0.2029541 | 0.053115 | 0.00000327 | 0.00014518 |
| Nitrosomonadales | 0.1113704 | 0.00461 | 0.00000719 | 0.000219295 |
| 258ds10 | 0 | 0.0427993 | 0.000082 | 0.000948273 |
| A21b | 0.3383511 | 0.1894143 | 0.0001685 | 0.001581308 |
| HTCC2188 | 0.00735 | 0.0682594 | 0.000182 | 0.001586 |
| Sphingobacteriales | 0.0453679 | 0.2947664 | 0.000293 | 0.002383067 |
| Pirellulales | 0.4854787 | 0.2638192 | 0.0003784 | 0.0028853 |
| JH-WHS99 | 0.00254 | 0.0575047 | 0.0006301 | 0.004521894 |
| Flavobacteriales | 0.00579 | 0.2901573 | 0.0007319 | 0.004699568 |
| PK329 | 0 | 0.1167653 | 0.0006965 | 0.004699568 |
| Opitutales | 0.0503145 | 0.166588 | 0.001053 | 0.0064233 |
| [Entotheonellales] | 0.5035694 | 0.2181667 | 0.001258 | 0.007308381 |
| Gemmatales | 0.8413552 | 0.3996796 | 0.001698 | 0.009006783 |
| Rubrobacterales | 0 | 0.0212899 | 0.00186 | 0.009023308 |
| CCM11a | 0.00254 | 0.0526761 | 0.001923 | 0.009023308 |
| IS-44 | 0 | 0.0783556 | 0.00186 | 0.009023308 |
| Methylophilales | 0.00367 | 0.0513592 | 0.002635 | 0.011906296 |
| Ellin7246 | 0.0377359 | 0.0068 | 0.003039 | 0.013241357 |
| Neisseriales | 0.00763 | 0.0928415 | 0.005125 | 0.020841667 |
| Thiotrichales | 0.0708078 | 0.2096068 | 0.007308 | 0.028760516 |
| N1423WL | 0.1363863 | 0.0814284 | 0.01027 | 0.037967879 |
| [Fimbriimonadales] | 0.0204933 | 0.1257641 | 0.0112 | 0.039876571 |
| S0208 | 0 | 0.0129495 | 0.01144 | 0.039876571 |
| Cytophagales | 0.1615436 | 0.6255281 | 0.01181 | 0.040022778 |
| A89 | 0.0130026 | 0.0654061 | 0.01227 | 0.040457838 |
| CCU21 | 0.0645892 | 0.1848052 | 0.0137 | 0.043984211 |
| CFB-26 | 0.0309519 | 0.126642 | 0.0154 | 0.048174359 |
| JG30-KF-AS9 | 0.9980934 | 0.5476115 | 0.01884 | 0.056060488 |
| Sva0725 | 0.0326479 | 0.0160223 | 0.01967 | 0.057136667 |
| C20 | 0 | 0.0445552 | 0.02671 | 0.072112609 |
| A31 | 0 | 0.0759413 | 0.02671 | 0.072112609 |
| Clostridiales | 0.0115893 | 0 | 0.02719 | 0.072112609 |
| Sphingomonadales | 0.3202605 | 0.7379037 | 0.02615 | 0.072112609 |
| NB1-j | 0.1346903 | 0.2506502 | 0.02872 | 0.074549787 |
| envOPS12 | 0.0196453 | 0.1553944 | 0.03032 | 0.077063333 |
| MND1 | 0.6832036 | 1.1595316 | 0.03228 | 0.080370612 |
| C114 | 0.0368879 | 0.1485904 | 0.03486 | 0.0850584 |
| Chromatiales | 0.00311 | 0.019534 | 0.03733 | 0.089299216 |
| Caulobacterales | 0.0375946 | 0.0704542 | 0.05083 | 0.114838148 |
| WCHB1-50 | 0.00735 | 0 | 0.05307 | 0.115616786 |
| agg27 | 0.0846584 | 0.0583826 | 0.0527 | 0.115616786 |
| [Saprospirales] | 0.3230872 | 0.5440997 | 0.05856 | 0.123177931 |
| Rhodocyclales | 0 | 0.0144859 | 0.06066 | 0.123342 |
| [Marinicellales] | 0 | 0.0129495 | 0.06066 | 0.123342 |
| S-BQ2-57 | 0.2134128 | 0.14025 | 0.06403 | 0.12806 |
| mle1-8 | 0.0185146 | 0.00768 | 0.09565 | 0.188214516 |
| CL500-15 | 0.0115893 | 0 | 0.1017 | 0.190883077 |
| Pla1 | 0.00509 | 0 | 0.1017 | 0.190883077 |
| Methylacidiphilales | 0.00551 | 0 | 0.1017 | 0.190883077 |
| LD1-PA13 | 0.0320826 | 0.0805505 | 0.1205 | 0.222742424 |
| Oscillatoriales | 0 | 0.00812 | 0.1363 | 0.237551429 |
| RF32 | 0 | 0.009 | 0.1363 | 0.237551429 |
| MIZ46 | 0 | 0.00549 | 0.1363 | 0.237551429 |
| Alteromonadales | 0 | 0.0116326 | 0.1363 | 0.237551429 |
| Phycisphaerales | 0.0676985 | 0.040385 | 0.1563 | 0.264841667 |
| Ktedonobacterales | 1.0742719 | 0.2548204 | 0.1637 | 0.273580822 |
| 32-20 | 0.2258501 | 0.3035458 | 0.1904 | 0.2966125 |
| Ellin6537 | 0.00311 | 0 | 0.1945 | 0.2966125 |
| SM1D11 | 0.0158293 | 0 | 0.1945 | 0.2966125 |
| d113 | 0.00367 | 0 | 0.1945 | 0.2966125 |
| Legionellales | 0.0284079 | 0.0208509 | 0.1858 | 0.2966125 |
| I025 | 0.00594 | 0 | 0.1945 | 0.2966125 |
| Anaeroplasmatales | 0.00325 | 0 | 0.1945 | 0.2966125 |
| H39 | 0.0278426 | 0.0533345 | 0.1975 | 0.297469136 |
| mle1-48 | 0.00155 | 0.0120716 | 0.2055 | 0.302060241 |
| B07WMSP1 | 0.629921 | 0.5713157 | 0.2201 | 0.315781395 |
| Gemmatimonadales | 0.00283 | 0.01273 | 0.2226 | 0.315781395 |
| WD2101 | 0.5312706 | 0.3843157 | 0.2201 | 0.315781395 |
| Chlamydiales | 0.0313759 | 0.0190951 | 0.2764 | 0.383190909 |
| PK29 | 0.2596287 | 0.2379202 | 0.2891 | 0.396294382 |
| DS-18 | 0.2209034 | 0.1927066 | 0.3085 | 0.410387234 |
| Nostocales | 0 | 0.00263 | 0.3162 | 0.410387234 |
| BD7-3 | 0 | 0.00505 | 0.3162 | 0.410387234 |
| GN03 | 0 | 0.00263 | 0.3162 | 0.410387234 |
| wb1H11 | 0 | 0.00373 | 0.3162 | 0.410387234 |
| SC-I-84 | 0.1855702 | 0.146615 | 0.3709 | 0.476313684 |
| MVS-40 | 0.00339 | 0 | 0.3851 | 0.479410204 |
| Herpetosiphonales | 0.0017 | 0 | 0.3851 | 0.479410204 |
| MVS-107 | 0.00212 | 0 | 0.3851 | 0.479410204 |
| Ellin5290 | 0.0261466 | 0.0160223 | 0.3953 | 0.487137374 |
| FAC88 | 0.0866371 | 0.0913052 | 0.5569 | 0.666096078 |
| Bdellovibrionales | 0.0202106 | 0.00922 | 0.5569 | 0.666096078 |
| Verrucomicrobiales | 0.0446612 | 0.0368732 | 0.5475 | 0.666096078 |
| [Roseiflexales] | 0.0450852 | 0.0607969 | 0.5657 | 0.670052427 |
| Rhodobacterales | 0.3560177 | 0.4696948 | 0.5747 | 0.674167308 |
| Ellin6067 | 0.1589996 | 0.1411279 | 0.5885 | 0.683780952 |
| 0319-7L14 | 0.1044451 | 0.146615 | 0.6025 | 0.693443396 |
| AKYG885 | 0.0360399 | 0.0496033 | 0.661 | 0.753663551 |
| Elusimicrobiales | 0.00749 | 0.00307 | 0.7194 | 0.8052 |
| CPla-3 | 0.00339 | 0.00219 | 0.7194 | 0.8052 |
| SBR1031 | 0.0368879 | 0.047189 | 0.8115 | 0.891918919 |
| IIb | 0.00678 | 0.00549 | 0.8728 | 0.942315044 |
| 11_24 | 0.6669503 | 0.6810575 | 0.9172 | 0.971162069 |
| Sva0853 | 0.00141 | 0.00219 | 0.9234 | 0.971162069 |
| p04_C01 | 0.0108826 | 0.0079 | 0.933 | 0.972871795 |
| Spirochaetales | 0.00806 | 0.00812 | 0.9526 | 0.984891525 |
| KD8-87 | 0.0282666 | 0.0487253 | 1 | 1 |
| Enterobacteriales | 0.011872 | 0.00527 | 1 | 1 |
