# Supplementary material for: Phytophthora Root Rot Modifies the Composition of the Avocado Rhizosphere Microbiome and Increases the Abundance of Opportunistic Fungal Pathogens
Source: Front Microbiol. 2021 Jan 12;11:574110. doi: 10.3389/fmicb.2020.574110 (PMC7835518; doi:10.3389/fmicb.2020.574110)
Supplement: Supplementary file 13 [file Table_6.docx]

Supplementary Material

**TABLE S6** Taxonomic composition and fold change at the *phylum* level of the rhizosphere fungal community between root rot asymptomatic and symptomatic avocado trees. ns: not significant

|  | **Relative abundance (%)** | |  |  |
| --- | --- | --- | --- | --- |
| **Phylum** | **Asymptomatic** | **Symptomatic** | **GFOLD(0.01)** | **log2fdc** |
| Ascomycota | 74.86724 | 71.02793124 | -0.0612161 | -0.075943 |
| Mortierellomycota | 12.37487664 | 18.11271468 | 0.517354 | 0.549585 |
| Basidiomycota | 8.676394567 | 6.417776047 | -0.388282 | -0.434938 |
| Glomeromycota | 1.031533437 | 2.427251414 | 1.13373 | 1.23419 |
| Rozellomycota | 1.294703698 | 1.440774903 | 0.0471734 | 0.154381 |
| **Others** | **Relative abundance (< 1%)** | |  |  |
| Aphelidiomycota | 0.011748672 | 0.018658684 | ns | ns |
| Basidiobolomycota | 0.008811504 | 0 | -0.545333 | -3.53428 |
| Blastocladiomycota | 0.020560177 | 0.040562357 | 0.246167 | 0.968216 |
| Chytridiomycota | 0.082240707 | 0.353703749 | 1.78055 | 2.09765 |
| Entomophthoromycota | 0.014685841 | 0 | -1.34598 | -4.23472 |
| Kickxellomycota | 1.429813431 | 0.090859679 | -3.65511 | -3.96381 |
| Mucoromycota | 0.126885662 | 0.019469931 | -1.99912 | -2.65198 |
| Neocallimastigomycota | 0.035246017 | 0.030016144 | ns | ns |
| Olpidiomycota | 0 | 0.008923718 | 0.973964 | 4.05068 |
| Zoopagomycota | 0.025259646 | 0.01135746 | -0.113029 | -1.08683 |
