# Supplementary material for: Phytophthora Root Rot Modifies the Composition of the Avocado Rhizosphere Microbiome and Increases the Abundance of Opportunistic Fungal Pathogens
Source: Front Microbiol. 2021 Jan 12;11:574110. doi: 10.3389/fmicb.2020.574110 (PMC7835518; doi:10.3389/fmicb.2020.574110)
Supplement: Supplementary file 14 [file Table_7.docx]

Supplementary Material

**TABLE S7** Taxonomic composition and fold change at the class level of the rhizosphere fungal community between root rot asymptomatic and symptomatic avocado trees. ns: not significant

|  | **Relative abundance (%)** | |  |  |
| --- | --- | --- | --- | --- |
| **Class** | **Asymptomatic** | **Symptomatic** | **GFOLD(0.01)** | **log2fdc** |
| Eurotiomycetes | 41.4399173 | 11.3785522 | -1.8337 | -1.86462 |
| Leotiomycetes | 13.2002209 | 22.8138918 | 0.759323 | 0.789338 |
| Mortierellomycetes | 12.3625405 | 18.0786423 | 0.516051 | 0.548307 |
| Dothideomycetes | 6.2197472 | 23.0126473 | 1.8493 | 1.88741 |
| Sordariomycetes | 2.6123173 | 5.5383842 | 1.01949 | 1.08402 |
| Agaricomycetes | 4.4409982 | 3.6619695 | -0.215168 | -0.278135 |
| Pezizomycetes | 3.0605292 | 2.7768989 | -0.0664996 | -0.140162 |
| Tremellomycetes | 3.3319235 | 1.1081636 | -1.48723 | -1.58738 |
| Glomeromycetes | 0.9722026 | 2.261757 | 1.11406 | 1.21776 |
| Not assigned | 10.4962639 | 4.4407668 | -1.18919 | -1.24081 |
| **Others** | **Relative abundance (< 1%)** | |  |  |
| Agaricostilbomycetes | 0.261408 | 0.0275824 | -2.65937 | -3.2059 |
| Aphelidiomycetes | 0.0117487 | 0.0186587 | ns | ns |
| Archaeorhizomycetes | 0.3971051 | 0.0162249 | -3.87444 | -4.54498 |
| Archaeosporomycetes | 0 | 0.1200646 | 4.92672 | 7.68488 |
| Atractiellomycetes | 0.2625828 | 0.5200094 | 0.77924 | 0.98479 |
| Basidiobolomycetes | 0.00881 | 0 | -0.545333 | -3.53428 |
| Blastocladiomycetes | 0.0205602 | 0.0405624 | 0.246167 | 0.968216 |
| Cystobasidiomycetes | 0 | 0.00973 | 1.11653 | 4.16616 |
| Endogonomycetes | 0 | 0.0113575 | 1.36617 | 4.37261 |
| Entomophthoromycetes | 0.0146858 | 0 | -1.34598 | -4.23472 |
| Exobasidiomycetes | 0.0428827 | 0.00811 | -1.11521 | -2.28431 |
| Geminibasidiomycetes | 0 | 0.0511086 | 3.67109 | 6.46572 |
| Geoglossomycetes | 0 | 1.2298506 | 8.29921 | 11.0327 |
| GS19 | 0.0152733 | 0.0527311 | 1.02021 | 1.75522 |
| Laboulbeniomycetes | 0.0187979 | 0.0146024 | ns | ns |
| Lecanoromycetes | 0.0804784 | 0.8509982 | 3.10011 | 3.39336 |
| Malasseziomycetes | 0 | 0.0365061 | 3.16956 | 5.98928 |
| Microbotryomycetes | 0.1057381 | 0.1257433 | ns | ns |
| Mucoromycetes | 0.1268857 | 0.00811 | -2.7512 | -3.8364 |
| Neocallimastigomycetes | 0.035246 | 0.0300161 | ns | ns |
| Olpidiomycetes | 0 | 0.00892 | 0.973964 | 4.05068 |
| Orbiliomycetes | 0.2531839 | 0.413736 | 0.489124 | 0.708008 |
| Paraglomeromycetes | 0.0129235 | 0 | -1.14829 | -4.05785 |
| Pucciniomycetes | 0.0757789 | 0.0511086 | -0.0561326 | -0.556652 |
| Rhizophydiomycetes | 0.013511 | 0.1297995 | 2.5329 | 3.21167 |
| Rozellomycotina incertae sedis | 0.00587 | 0.5094632 | 5.24678 | 6.3032 |
| Saccharomycetes | 0.0481696 | 0.2628441 | 2.03692 | 2.43497 |
| Spizellomycetes | 0.0264345 | 0.0438073 | 0.054611 | 0.723513 |
| Xylonomycetes | 0 | 0.3253101 | 6.37581 | 9.11677 |
| Zoopagomycetes | 0.0252596 | 0.0113575 | -0.113029 | -1.08683 |
