# Supplementary material for: Phytophthora Root Rot Modifies the Composition of the Avocado Rhizosphere Microbiome and Increases the Abundance of Opportunistic Fungal Pathogens
Source: Front Microbiol. 2021 Jan 12;11:574110. doi: 10.3389/fmicb.2020.574110 (PMC7835518; doi:10.3389/fmicb.2020.574110)
Supplement: Supplementary file 15 [file Table_8.docx]

Supplementary Material

**TABLE S8** Taxonomic composition and fold change at the order level of the rhizosphere fungal community between root rot asymptomatic and symptomatic avocado trees. ns: not significant

|  | **Relative abundance (%)** | |  |  |
| --- | --- | --- | --- | --- |
| **Order** | **Asymptomatic** | **Symptomatic** | **GFOLD(0.01)** | **log2fdc** |
| Helotiales | 13.1450021 | 19.8390486 | 0.562781 | 0.593823 |
| Mortierellales | 12.3625405 | 18.0786423 | 0.516051 | 0.548307 |
| Onygenales | 23.885051 | 3.0275743 | -2.92256 | -2.97952 |
| Pleosporales | 1.6871094 | 14.0913627 | 2.99468 | 3.06177 |
| Chaetothyriales | 7.3235349 | 4.2987985 | -0.713606 | -0.76845 |
| Venturiales | 1.3828187 | 6.0648835 | 2.05365 | 2.13245 |
| Pezizales | 3.0605292 | 2.7768989 | -0.0664996 | -0.140162 |
| Capnodiales | 1.530852 | 1.8626234 | 0.186926 | 0.283076 |
| Magnaporthales | 0.6972837 | 2.5740871 | 1.77024 | 1.88348 |
| Eurotiales | 0.7525025 | 2.475115 | 1.60616 | 1.71707 |
| Glomerales | 0.964566 | 2.1692748 | 1.06412 | 1.16892 |
| Xylariales | 1.3740072 | 1.6573779 | 0.168865 | 0.270602 |
| Agaricales | 1.5525871 | 1.3004291 | -0.149529 | -0.255332 |
| Verrucariales | 2.0689412 | 0.2149805 | -3.05416 | -3.26159 |
| Thelebolales | 0.0323088 | 2.0954513 | 5.5694 | 5.99375 |
| Trichosporonales | 1.880375 | 0.1387233 | -3.49948 | -3.75278 |
| Not assigned | 19.9439588 | 7.1600672 | -1.43784 | -1.47779 |
| **Others** | **Relative abundance (< 1%)** | |  |  |
| Acarosporales | 0 | 0.0673335 | 4.07865 | 6.85803 |
| Acrospermales | 0 | 0.0648998 | 4.02438 | 6.80557 |
| Agaricostilbales | 0.261408 | 0.0275824 | -2.65937 | -3.2059 |
| Archaeorhizomycetales | 0.3342497 | 0.0162249 | -3.62365 | -4.29679 |
| Archaeosporales | 0 | 0.1200646 | 4.92672 | 7.68488 |
| Atheliales | 0.00999 | 0.0170362 | ns | 0.755222 |
| Atractiellales | 0.2625828 | 0.5200094 | 0.77924 | 0.98479 |
| Auriculariales | 0.00764 | 0.0454298 | 1.51954 | 2.49125 |
| Basidiobolales | 0.00881 | 0 | -0.545333 | -3.53428 |
| Blastocladiales | 0.0205602 | 0.0405624 | 0.246167 | 0.968216 |
| Boletales | 0.0687297 | 0.062466 | ns | -0.131525 |
| Botryosphaeriales | 0.2449598 | 0.1233096 | -0.673966 | -0.984256 |
| Cantharellales | 0.1028009 | 0.5045957 | 2.00971 | 2.28937 |
| Chaetosphaeriales | 0.0605057 | 0.0137912 | -1.27616 | -2.0648 |
| Corticiales | 0 | 0.0600323 | 3.9093 | 6.69453 |
| Cystofilobasidiales | 0 | 0.01298 | 1.57963 | 4.55318 |
| Diversisporales | 0 | 0.0243374 | 2.55683 | 5.41991 |
| Dothideales | 0.1104375 | 0.2271492 | 0.724763 | 1.0379 |
| Endogonales | 0 | 0.0113575 | 1.36617 | 4.37261 |
| Entomophthorales | 0.0146858 | 0 | -1.34598 | -4.23472 |
| Entylomatales | 0.0164481 | 0.00811 | ns | -0.932834 |
| Erysiphales | 0 | 0.4218485 | 6.75222 | 9.49086 |
| Exobasidiales | 0.0205602 | 0 | -1.85962 | -4.70421 |
| Filobasidiales | 0.4405752 | 0.0957272 | -1.87287 | -2.19214 |
| Geastrales | 0.1116124 | 0 | -4.35957 | -7.11171 |
| Geminibasidiales | 0 | 0.0511086 | 3.67109 | 6.46572 |
| Geoglossales | 0 | 1.2298506 | 8.29921 | 11.0327 |
| Gigasporales | 0 | 0.0681448 | 4.0963 | 6.87511 |
| Glomerellales | 0.0146858 | 0.1054621 | 2.12621 | 2.7987 |
| Gomphales | 0.00646 | 0 | -0.0425328 | -3.11925 |
| GS11 | 0.00587 | 0.5094632 | 5.24678 | 6.3032 |
| GS19 | 0.0152733 | 0.0527311 | 1.02021 | 1.75522 |
| Hymenochaetales | 0.0146858 | 0.0381286 | 0.555864 | 1.35024 |
| Hypocreales | 0.3348372 | 0.410491 | 0.0888796 | 0.294211 |
| Kriegeriales | 0.1057381 | 0 | -4.28081 | -7.03413 |
| Lecanorales | 0 | 0.4518647 | 6.85172 | 9.58984 |
| Malasseziales | 0 | 0.0365061 | 3.16956 | 5.98928 |
| Microbotryales | 0 | 0.0113575 | 1.36617 | 4.37261 |
| Microbotryomycetes incertae sedis | 0 | 0.0868034 | 4.45204 | 7.2206 |
| Microstromatales | 0.00587 | 0 | ns | -2.99372 |
| Mucorales | 0.1268857 | 0.00811 | -2.7512 | -3.8364 |
| Mycocaliciales | 0 | 0.0121687 | 1.47676 | 4.46572 |
| Neocallimastigales | 0.035246 | 0.0300161 | ns | -0.217094 |
| Olpidiales | 0 | 0.00892 | 0.973964 | 4.05068 |
| Orbiliales | 0.2267494 | 0.413736 | 0.64148 | 0.866705 |
| Ostropales | 0.0340711 | 0.2466191 | 2.3807 | 2.83574 |
| Paraglomerales | 0.0129235 | 0 | -1.14829 | -4.05785 |
| Platygloeales | 0 | 0.0186587 | 2.14863 | 5.05068 |
| Polyporales | 0.1732929 | 0.2766353 | 0.40819 | 0.674115 |
| Pucciniales | 0.0757789 | 0.0324499 | -0.623986 | -1.1991 |
| Pyxidiophorales | 0.0187979 | 0.0146024 | ns | -0.330751 |
| Rhizophydiales | 0.013511 | 0.1297995 | 2.5329 | 3.21167 |
| Russulales | 0.0405329 | 0.0567873 | ns | 0.48618 |
| Saccharomycetales | 0.0481696 | 0.2628441 | 2.03692 | 2.43497 |
| Sebacinales | 0.168006 | 0.5865317 | 1.57008 | 1.80065 |
| Sordariales | 0.1139621 | 0.7098412 | 2.37417 | 2.63317 |
| Spizellomycetales | 0.0264345 | 0.0438073 | 0.054611 | 0.723513 |
| Sporidiobolales | 0 | 0.0275824 | 2.74714 | 5.595 |
| Symbiotaphrinales | 0 | 0.3253101 | 6.37581 | 9.11677 |
| Teloschistales | 0.00822 | 0 | -0.434737 | -3.44117 |
| Thelephorales | 0.4570234 | 0.0949159 | -1.93766 | -2.25712 |
| Trapeliales | 0.0158607 | 0 | -1.46426 | -4.34164 |
| Trechisporales | 0.2960665 | 0.1525145 | -0.671491 | -0.952182 |
| Tremellales | 0.8523662 | 0.8347733 | ns | -0.029682 |
| Tubeufiales | 0.9868885 | 0.3399125 | -1.35512 | -1.53514 |
| Zoopagales | 0.0252596 | 0.0113575 | -0.113029 | -1.08683 |
